# Proteomic profile of culture filtrate from the Brazilian vaccine strain *Mycobacterium bovis *BCG Moreau compared to *M. bovis *BCG Pasteur

**DOI:** 10.1186/1471-2180-11-80

**Published:** 2011-04-20

**Authors:** Marcia Berrêdo-Pinho, Dario E Kalume, Paloma R Correa, Leonardo HF Gomes, Melissa P Pereira, Renata F da Silva, Luiz RR Castello-Branco, Wim M Degrave, Leila Mendonça-Lima

**Affiliations:** 1 Laboratório de Genômica Funcional e Bioinformática, Instituto Oswaldo Cruz, FIOCRUZ, Avenida Brasil, 4365, Manguinhos, CEP 21040 -900 Rio de Janeiro, RJ, Brazil; 2 Laboratório Interdisciplinar de Pesquisas Médicas, Instituto Oswaldo Cruz, FIOCRUZ, Avenida Brasil, 4365, Manguinhos, CEP 21040 -900 Rio de Janeiro, RJ, Brazil; 3 Centro de Pesquisas Arlindo de Assis, Fundação Ataulpho de Paiva (FAP), Rio de Janeiro, RJ, Brazil

## Abstract

**Background:**

Bacille Calmette-Guerin (BCG) is currently the only available vaccine against tuberculosis (TB) and comprises a heterogeneous family of sub-strains with genotypic and phenotypic differences. The World Health Organization (WHO) affirms that the characterization of BCG sub-strains, both on genomic and proteomic levels, is crucial for a better comprehension of the vaccine. In addition, these studies can contribute in the development of a more efficient vaccine against TB. Here, we combine two-dimensional electrophoresis (2DE) and mass spectrometry to analyse the proteomic profile of culture filtrate proteins (CFPs) from *M. bovis *BCG Moreau, the Brazilian vaccine strain, comparing it to that of BCG Pasteur. CFPs are considered of great importance given their dominant immunogenicity and role in pathogenesis, being available for interaction with host cells since early infection.

**Results:**

The 2DE proteomic map of *M. bovis *BCG Moreau CFPs in the pH range 3 - 8 allowed the identification of 158 spots corresponding to 101 different proteins, identified by MS/MS. Comparison to BCG Pasteur highlights the great similarity between these BCG strains. However, quantitative analysis shows a higher expression of immunogenic proteins such as Rv1860 (BCG1896, Apa), Rv1926c (BCG1965c, Mpb63) and Rv1886c (BCG1923c, Ag85B) in BCG Moreau when compared to BCG Pasteur, while some heat shock proteins, such as Rv0440 (BCG0479, GroEL2) and Rv0350 (BCG0389, DnaK), show the opposite pattern.

**Conclusions:**

Here we report the detailed 2DE profile of CFPs from *M. bovis *BCG Moreau and its comparison to BCG Pasteur, identifying differences that may provide relevant information on vaccine efficacy. These findings contribute to the detailed characterization of the Brazilian vaccine strain against TB, revealing aspects that may lead to a better understanding of the factors leading to BCG's variable protective efficacy against TB.

## Background

Tuberculosis (TB) remains a major cause of morbidity and mortality, particularly in developing countries, and is considered a serious public health problem worldwide, killing almost 2 million people every year [1]. According to the WHO, one-third of the world's population is infected with *Mycobacterium tuberculosis (Mtb)*. The incidence of new cases of TB has increased mainly due to the impact of the HIV epidemic [2] and the emergence of resistance to anti-TB drugs [3]. The currently available vaccine, *Mycobacterium bovis *bacillus Calmette-Guérin (BCG), is one of the oldest and most commonly administered vaccines worldwide [4]. It was obtained in the early 1920's by Albert Calmette and Camille Guérin at the Pasteur Institute, Lille, France, after 231 serial passages of a clinical isolate of *M. bovis *in glycerinated medium containing ox bile [5]. Attenuation during *in vitro *passages is believed to have resulted from the loss and/or reorganization of genomic regions, some of which have been recently identified [6-9]. *M. bovis *BCG Moreau is the strain used in Brazil for vaccine production since the 1930's [10]. According to recent molecular studies [11], it is considered an "old" strain, more similar to the original BCG derived by Calmette and Guérin. Vaccination with BCG has many advantages, yielding efficient protection against severe childhood forms of TB, and also against leprosy [12]. In addition, it is recognized as a safe and inexpensive vaccine that can be administered shortly after birth [13,14]. On the other hand, it shows variable protection against the most common form of the disease, pulmonary tuberculosis in adults, and it does not prevent the establishment of latent TB. It has been reported that different *M. bovis *BCG strains, including BCG Moreau, induce varying levels of protection against *M. tuberculosis *infection in animal models [15]. Comparative genetic analysis of BCG strains has revealed that each vaccine currently in use is unique [11], and providing several clues for the failure of BCG as an effective vaccine. Proteomic studies have shown that BCG strains with similar genomic content, such as BCG Denmark and Phipps, exhibit phenotypic differences that can be particularly important for pathogenesis, immune response and variable efficacy of BCG vaccine [16]. Other factors have also been implicated in its unpredictable efficacy: (i) the genetic variability amongst vaccinated individuals; (ii) cross-reactivity of the immune response to BCG due to environmental mycobacteria [17]; (iii) differences in vaccine production procedures, variable doses, and bacterial viability, amongst others [18,19]. New vaccination strategies are therefore urgently needed, particularly against pulmonary forms of TB.

The modulation of cellular functions of the host cell is a dynamic process that requires viable mycobacteria, supporting the idea that the components actively secreted by the living bacteria are the main players involved in this process [20]. Membrane and membrane associated proteins also play an important role in this process [21]. Subunit vaccines based on mixtures of culture filtrate proteins have resulted in protective immunity in animal models of TB [22-26]. These molecules are also strongly recognized during *Mtb *infection in various animal models, as well as in early stages of pulmonary TB in humans [27,28]. Culture filtrate is therefore an attractive source of potential candidate antigens for the development of new vaccines and diagnostic reagents. In this report, we have employed a combination of 2DE and mass spectrometry analysis in order to generate a proteomic map of CFPs from the Brazilian *M. bovis *BCG Moreau strain, comparing it to the reference strain, *M. bovis *BCG Pasteur. The data presented may contribute to the identification of useful markers for quality control of the BCG Moreau vaccine production, and yield possible clues regarding the variable effectiveness of these vaccine strains.

## Results

### Protein separation, identification and sub-cellular localization

**Figure 1:**
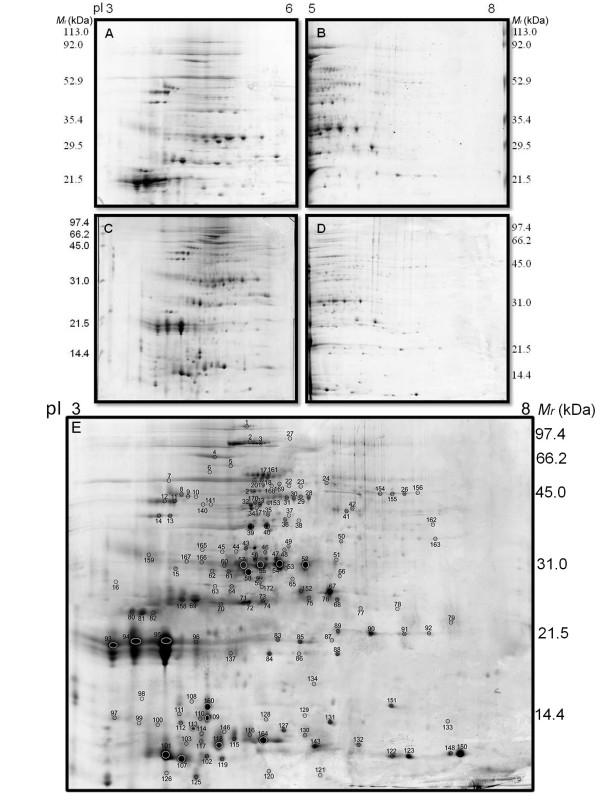
**2DE proteomic profile of CFPs from *M. bovis *BCG Moreau**. Proteins (500 ug) were applied to 17 cm IPG strips in the pH intervals of 3 - 6 (panels A and C) and 5 - 8 (panels B and D) and separated in the second dimension across 12% (panels A and B) and 15% (panels C and D) SDS-PAGE. The images were merged to obtain a composite map in the pH range 3 - 8 (panel E). Protein spots were visualized by colloidal CBB-G250 staining. Identified proteins are numbered in panel E and detailed in Additional file 2, Table S1. Molecular weight standards indicated in kDa.

**Figure 2:**
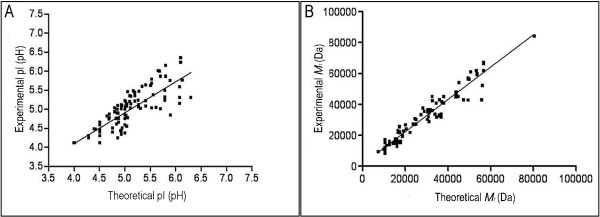
**Correlation between experimentally determined and theoretical pI and *M***_**r **_**and distribution of predicted cellular localization of the identified proteins**. The experimental and theoretical pI (panel A) and *M*_r _(panel B) values for the identified protein were compared. Overall, a positive Spearman correlation coefficient *r *= 0.75, *p *< 0.0001 and *r *= 0.95, *p *< 0.0001, is observed for the data in the pI range between 3 and 8 and *M*_r _range of 9 to 120 kDa, respectively.

The BCG strains were grown in static cultures, as surface pellicles, for 15 days, with no apparent difference in growth. The genetic profile of the 2 strains was confirmed by PCR (Additional file 1, Figure S1), corroborating with previous reports [29] The preliminary separation of BCG Moreau CFPs by 2DE revealed that most protein spots clustered in the pH range 3-8 (data not shown). To generate proteomic maps, samples were therefore applied to immobilized pH gradient (IPG) strips in the pH intervals 3-6 (Figure 1A and 1C) and 5-8 (Figure 1B and 1D) and subsequently separated in the second dimension across 12% (Figure 1A and 1B) and 15% SDS-PAGE (Figure 1C and 1D). To aid in visualization, gel images were merged to produce an artificial map representative of the pH range 3-8 (Figure 1E) comprising all the 280 spots resolved in the individual gels. These spots were excised and digested with trypsin. The resulting peptides were submitted to mass spectrometry analysis leading to the putative identification of 158 protein spots corresponding to 101 different proteins (Additional file 2, Table S1). For clarity, we have adopted the standard protein nomenclature used for *M. tuberculosis *strain H37Rv (http://genolist.pasteur.fr/tuberculist) and *M. bovis *BCG Pasteur 1173P2 (http://genolist.pasteur.fr/BCGList/). Identified proteins showed a pI variation between 3-8 and a molecular mass (*M*_r_) range between 9 and 120 kDa. The comparison of experimentally determined and theoretical *M*_r _and pI values of the identified protein spots from BCG Moreau against the predicted values for *M. tuberculosis *strain H37Rv proteins, obtained from the search with Mascot version 2.2, showed a positive correlation according to the Spearman coefficient (Figure 2A and 2B) Considering the fact that the proteins identified in this study were obtained from the culture filtrate, we analyzed the presence of possible signals that could direct these proteins to the extracellular fraction (Additional file 3, Table S2), using Signal P (for sec-dependent secretion; [30]), LipoP (lipoproteins; [31]), TatP (for secretion through the twin-arginine translocation system; [32]) and SecretomeP (for non-classical secretion of leaderless proteins; [33]). Of the 101 proteins, 67 (66%) have no extracellular prediction. However, when we compare our data to 2 previous reports on the culture filtrate proteome of *M. tuberculosis *H37Rv - the 2DE database at the Max Planck Institute (http://web.mpiib-berlin.mpg.de/) and a recent work by de Souza and collaborators [34] - 93 proteins (92%) have been previously reported in one or both studies, including 60 of the proteins with no extracellular prediction. We also evaluated the number of potential transmembrane (TM) domains using TMHMM ([35]; Additional file 3, Table S2). Thirteen proteins were found to contain 1 predicted TM domain which, although coinciding in all cases with the signal peptide region predicted by SignalP, does not exclude a possible membrane localization for some of these proteins [36]. For the 22 proteins with a predicted signal peptide, the theoretical pI and Mr were calculated for the full protein and for the mature protein, after removal of the signal peptide region predicted by SignalP (Additional file 4, Table S3).

### Predicted biological functions for the identified proteins

**Figure 3:**
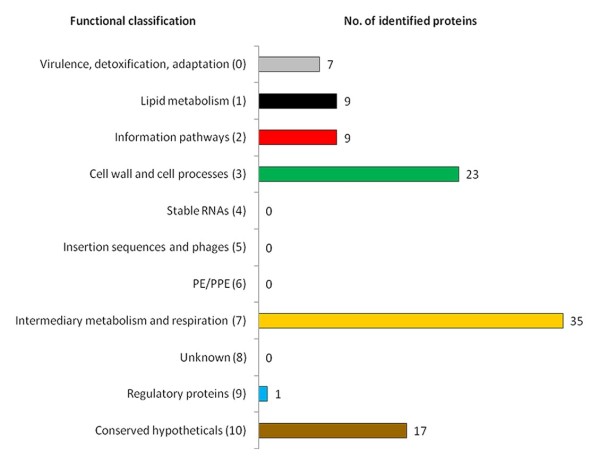
**Functional classification of the identified *M. bovis *BCG Moreau CFPs**. Identified proteins were classified into functional categories according to BCGList (http://genolist.pasteur.fr/BCGList/).

The assignment of the identified CFPs into functional categories was based on the functional classification tree from BCGList (http://genolist.pasteur.fr/BCGList/). The 101 proteins identified by MS/MS are distributed across 7 of those functional groups (Figure 3). The largest groups were "intermediary metabolism and respiration" (35%), "cell wall and cell processes" (23%) and "conserved hypotheticals" (17%).

### Differential CFP proteomic profiles between *M. bovis *BCG strains Moreau and Pasteur

**Figure 4:**
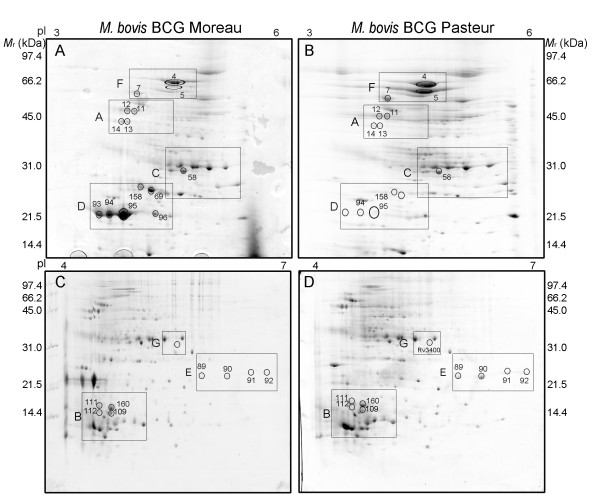
**Comparative 2DE profiles of CFPs from *M. bovis *BCG strains Moreau and Pasteur**. Proteins (500 ug) were applied to IPG strips in the pH intervals of 3 - 6 (panels A and B) and 4 - 7 (panels C and D) and separated in the second dimension in 12% (panels A and B) and 15% (panels C and D) SDS-PAGE. Protein spots were visualized by colloidal CBB-G250 staining and the gels images compared with PDQuest (Bio-Rad). Molecular weight standards indicated in kDa. The sectors shown in more detail in Additional files 5 and 6, Figures S2 and S3, are indicated in the figure (sectors A - G).

**Figure 5:**
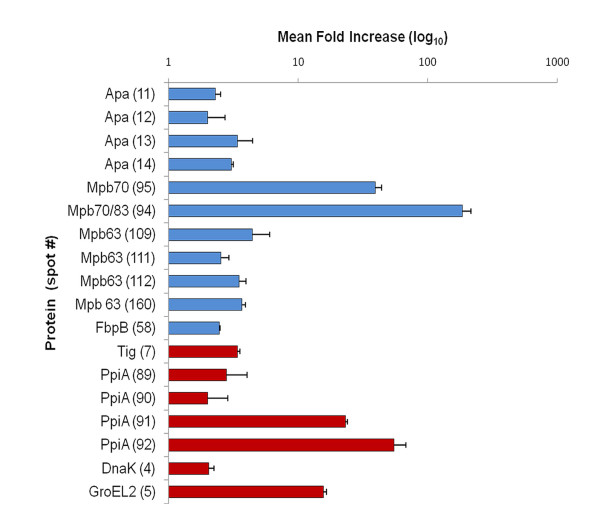
**CFPs differentially expressed between BCG strains Moreau and Pasteur**. Bars represent fold increase (mean ± SD of the pixel intensity ratios for each specified protein spot between strains). Protein spots more expressed in BCG Moreau compared to Pasteur are represented by blue bars while those more expressed in BCG Pasteur compared to Moreau are represented by red bars. Individual values are detailed in Table 1.

The 2DE profiles from *M. bovis *BCG strains Moreau and Pasteur were compared to identify differences that could provide relevant information about the Brazilian vaccine strain. For quantification analyses of the protein spots derived from both strains, the PDQuest software was used, comparing the optical densities of the matched spots in 2DE gel images. The experiments were repeated at least 3 times, and only the differences confirmed in all comparisons were accepted as strain specific. As expected, the proteomic profiles of CFPs from BCG strains Moreau and Pasteur were very similar (Figure 4 A-D); however, some variations in relative protein quantifications were observed. A total of 9 proteins represented by 18 spots showed a differential expression pattern between the two BCG strains (Table 1, Figure 5 and Additional file 5, Figure S2). In addition, 2 proteins were found exclusively in BCG Moreau and one protein exclusively in BCG Pasteur (Figure 4 A-D and Additional file 6, Figure S3).

**Table 1:** CFPs differentially expressed between BCG strains Moreau and Pasteur

Spot number	*Mtb *ortholog	BCG Pasteur ortholog	Protein	**Ratio**^**#**^	**Fold Increase**^**## **^**± SD**	*p*-value
11^###^	Rv1860	BCG1896	Apa	M/P	2.31 ± 0.22	0.09
12^###^				M/P	2.01 ± 0.71	0.27
13				M/P	3.42 ± 1.06	0.02
14				M/P	3.05 ± 0.11	0.009
95	Rv2875	BCG2897	Mpt70	M/P	39.50 ± 4.52	0.0004
94	Rv2875/Rv2873	BCG2897/BCG2895	Mpt70/Mpt83	M/P	185.27 ± 30.35	0.004
109^###^		BCG1965c		M/P	4.45 ± 1.59	0.19
111				M/P	2.54 ± 0.39	0.03
112^###^	Rv1926c		Mpt63	M/P	3.50 ± 0.48	0.41
160				M/P	3.68 ± 0.23	0.03
58	Rv1886c	BCG1923c	FbpB	M/P	2.46 ± 0.034	0.01
7	Rv2462c	BCG2482c	Tig	P/M	3.42 ± 0.13	0.001
89^###^		BCG0009		P/M	2.81 ± 1.24	0.07
90	Rv0009		PPIase A	P/M	2.01 ± 0.87	0.008
91				P/M	23.28 ± 0.87	0.005
92				P/M	55.21 ± 12.61	0.05
4	Rv0350	BCG0389	DnaK	P/M	2.04 ± 0.21	0.03
5	Rv0440	BCG0479	GroEL2	P/M	15.66 ± 0.93	0.00005

^#^In order to report values as fold increase, ratio was calculated for BCG Moreau (M) in relation to Pasteur (P) or vice-versa, as specified

^##^Ratio of mean pixel intensity value (±SD) for the specified protein spot in one BCG strain vs. the other

^###^Protein spots that did not show statistically significant change (p > 0.05)

Quantitative analysis revealed that 5 proteins were present in at least 2-fold higher concentration in BCG Moreau when compared to BCG Pasteur (Additional file 5, Figure S2): the Apa glycoprotein (Rv1860/BCG1896; spots 11, 12, 13 and 14); the immunogenic protein MPB63 (Rv1926c/BCG1965c; spots 109,111, 112 and 160); the secreted antigen 85B (Ag85B, FbpB, Rv1886c/BCG1923c; spot 58); and proteins MPB70 and MPB83 (Rv2875/BCG2897 and Rv2873/BCG2985; spots 94 and 95, respectively) (Table 1 and Figure 5). Spot 93 was also identified as MPB70 but was observed only in BCG Moreau (Figure 4).

Four proteins were more expressed in BCG Pasteur when compared to Moreau (Additional file 5, Figure S2): the heat shock proteins Hsp70 (DnaK, Rv0350/BCG0389; spot 4) and Hsp65 (GroEL2, Cpn60.2, Rv0440/BCG0479; spot 5); the presumed trigger factor (Tig, Rv2462c/BCG2482c; spot 7) and the probable iron-regulated peptidyl-prolyl cis-trans isomerase A (PPIaseA, Rv0009/BCG0009; spots 89, 90, 91 and 92) (Table 1 and Figure 5).

As expected, MPB64 (Rv1980c, spots 69 and 158) and CFP21 (Rv1984c; spot 96) were identified in BCG Moreau but were not present in BCG Pasteur (Figure 4 and Additional file 6, Figure S3) due to the loss of genomic region RD2 in the more recent BCG strains [7]. On the other hand, BCG Moreau contains a genomic deletion (RD16) encompassing genes *rv3400-rv3405c *(*bcg3470-bcg3475c*). In this study we identified only one protein present in BCG Pasteur and absent in BCG Moreau: a probable hydrolase encoded by *rv3400 *(*bcg3470*) (Figure 4 and Additional file 6, Figure S3). This difference is consistent with previous reports [7].

## Discussion

The main goal of this study was to perform a comprehensive proteomic analysis of CFPs from *M. bovis *BCG strains Moreau and Pasteur by combining 2DE and mass spectrometry, allowing for the evaluation of phenotypic differences between the two strains. The 2DE patterns were highly similar, presenting numerous prominent common spots that could be used as landmarks. From 2DE gels of CFP preparations from *M. bovis *BCG Moreau, 158 spots were identified. The *M*_r _and pI values estimated by 2DE showed a good correlation with expected values; however 34% of the identified proteins were detected in 2 or more spots with different *M*_r _and/or pI. This is probably due to post-translational modifications (PTMs) such as glycosylation, phosphorylation or other modifications already described for several of the identified proteins [37-40]. For example, Rv1827 (BCG1862, Cfp17, GarA; spots 80, 81, 82) and Rv0020c (BCG0050c, TB39.8. FhaA; spots 8, 9 and 10) possess FHA domains that bind phosphothreonine [40], and Rv0685 (BCG0734, Tuf; spots 28, 29, 30 and 31) is also described as being phosphorylated in the same amino acid residues [38].

Protein modifications in prokaryotes are of great biological interest but are not yet well understood. In this work we observed several deaminated proteins (approximately 22%), possibly associated with important biological processes such as protein turnover, molecular aging and cell adhesion [41]. In addition, deamination may be useful for the refinement of protein searches by MS/MS as well as tryptophan oxidation and N-terminal pyroglutamylation [42], which are also observed in several peptides identified in this study (Additional file 2, Table S1). Interestingly, formylation was only observed for one N-terminal methionine residue in Rv1827 (BCG1862, Cfp17, GarA), a FHA domain-containing protein that constitutes the major substrate for an essential kinase, PknB, in *Mtb *cell extracts [43]. Formylated peptides and proteins are specific signatures of bacterial metabolism, and attractive targets to the innate immune system, serving as potent chemoattractants for mammalian phagocytic leukocytes [44]. The lack of other proteins showing this particular PTM could also indicate that peptide deformylases are operating with high efficiency. Another chemical modification observed was threonine acetylation. Although N-terminal acetylation is common in eukaryotic proteins, it has been reported to be rare in prokaryotes [45]. This PTM is present in 2 proteins identified as putative ESAT-6 like proteins, EsxI (Rv1037c, BCG1095c) and EsxN (Rv1793, BCG1825) (Additional file 2, Table S1). The N-terminal acetylation may not always alter function, but in *Mtb *it has been shown that antigen ESAT-6, which normally interacts with the protein CFP-10, fails to do so when acetylated [46], possibly hindering its secretion via the mycobacterial-specific type VII secretion system [47].

In the current study, only 21 (21%) of the identified proteins were found to have a predicted signal peptide. Of these, 13 have one predicted TM segment coinciding with the predicted signal peptide region. Since current methods are not fully efficient in predicting membrane retention of proteins with typical Sec-type signal peptides [36], we cannot discard the possibility that the extracellular localization of some of these proteins may result from the gradual proteolytic release of membrane proteins containing a single amino-terminal membrane anchor.

Several mycobacterial proteins that do not present a canonical signal peptide can be secreted by alternative secretion mechanisms, such as the twin-arginine translocation system, the alternative SecA2 pathway or the recently described Type VII (Esx) secretion system [48-50]. Other studies on the culture filtrate proteome of mycobacteria have also reported the presence of numerous leaderless proteins [51-53]. Some of the proteins identified by us are also reported in the membrane proteome of BCG Moreau [54] and the cell wall proteome of *M. smegmatis *[55]. The abundance in the culture filtrate of *M. bovis *BCG Moreau of proteins without signal peptide prediction may also result from bacterial lysis, in combination with high levels of protein expression and extracellular stability, as described for several *Mtb *proteins [56]. Nevertheless, the precise mechanism by which these proteins are exported is still unknown.

Approximately 24% of the CFPs identified in the present study have no defined function (conserved hypotheticals); among these we can highlight the conserved hypothetical proteins TB27.3 (Rv0577, BCG0622), TB18.6 (Rv2140c, BCG2175c), Rv2626c (BCG2653c) and TB15.3 (Rv1636, BCG1674) this last, recently described as being differentially expressed in the secretome of a recombinant BCG strain [57]. Although their functions have not been established, these proteins have been considered as antigens, able to distinguish between tuberculosis clinical states, or attractive targets for the development of new drugs, vaccines and diagnostic strategies for TB [58-60].

Several other mycobacterial antigens, previously described as important for vaccine development and TB diagnosis, have also been identified in the present study, including the ESAT-6 like protein EsxG (Rv0287, BCG0327), recognized by multiple T-cell lines and able to boost IFN-γ levels in mouse and guinea pig models of TB [61], and the secreted MPT51 protein (Rv3803c, BCG3865c), described as being able to induce higher levels of antigen-specific CD8^+ ^T-cell responses [62].

Proteins involved in biosynthesis and degradation of fatty acids were also identified, such as the members of the antigen-85 complex, FbpA (Rv3804c, BCG3866c), FbpB (Rv1886c, BCG1923c), FbpC (Rv0129c, BCG0163) and FbpD (Rv3803c, BCG3865c; Mpb51), essential for the biosynthesis of mycolic acids [63]. In this work, Ag85B (FbpB) was found to be more abundant in the culture filtrate of BCG Moreau than in that of BCG Pasteur. The protein has been shown to induce partial protection against TB in animal models, and is considered an important immunodominant antigen and a promising vaccine candidate [64]. MPT64 (Rv1980) and CFP21 (Rv1984c) were found only in BCG Moreau; both are described in the literature as immunogenic antigens and potential targets for the development of new vaccines against TB [65,66]. The genes encoding these proteins are located in RD2, a genomic region deleted in a number of more recent BCG strains, including *M. bovis *BCG Pasteur, but present in BCG Moreau [6,7].

The low levels of MPB70 and MPB83 in *M. bovis *BCG Pasteur were also confirmed in our study. Their reduced expression is due to a point mutation in the translational start codon of the *sigK *gene [67], observed in many BCG strains, but absent in BCG Moreau. Immunologic studies have shown that both proteins induce cellular and humoral immune responses in experimental models of infection and in natural infection in humans [68,69]. MPB63 is a protein only found in species within the *Mtb *complex [70], shown to be immunodominant both in humans and animal models [71] and a promising candidate for serodiagnosis of active TB as well as for vaccine development. MPB63 was identified in four different spots (109,111,112 and 160), 2 of which (111 and 160) showed statistically significant differences in expression, with an increase of more than 3-fold in BCG Moreau as compared to BCG Pasteur (Table 1 and Figure 5). These 4 protein spots are likely to represent isoforms, probably differing due to the presence of PTMs, known to cause changes in pI resulting in slightly different migration. Moreover, MPB63 contains an N-terminal signal sequence as predicted by the SignalP software, which was experimentally verified [72].

The alanine-proline rich protein (Apa, Rv1860, BCG1896, spots 11, 12, 13 and 14) is known to present a high content of proline and carbohydrate groups [37] that interferes with its migratory behavior in SDS-PAGE. Although we have not identified modifications such as glycosylation, this protein displays a characteristic four-spot pattern (doublet of 2 horizontally dispersed spots) on 2DE [39] (Additional file 5, Figure S2). The isoforms of lowest molecular mass (spots 13 and 14) showed a statistically significant 3-fold increase in expression in BCG Moreau (Table 1 and Figure 5). This protein seems to be restricted to the *Mtb *complex and has been shown to be a target for immune recognition in animals immunized with live BCG [73]. In addition to its high immunogenicity, it has also been described as a potential adhesin involved in the colonization of target cells [39]. Its higher expression could contribute to an increase in the immunogenicity of BCG Moreau.

Four proteins were found to be at least 2-fold more expressed in BCG Pasteur compared to Moreau: a peptidyl-prolyl cis-trans isomerase (PPIaseA, Rv0009, BCG0009), the trigger factor (TF, Rv2462c, BCG2482c), Hsp65 (GroEL2, Rv0440, BCG0479) and Hsp70 (DnaK, Rv0350, BCG0389), all described as participating in protein folding and response to stress, among other functions. The gene *ppiA *is located in the Pasteur-specific DU1 duplicated region. The duplication of this gene alone may be responsible for the observed increased expression of PPIaseA in BCG Pasteur. Comparative transcriptome analysis has shown that *bcg0009, bcg0389, bcg0479 *and *bcg2482c *are all up-regulated in BCG Pasteur when compared to BCG Tokyo [11]. Considering the genealogy of BCG vaccines [7], BCG Moreau, Tokyo and Russia belong to the same group of "older" strains, closer to the original attenuated strain derived by Calmette and Guérin in the early 1920's, and all lack the DU1 duplication. The genome of BCG Pasteur, unlike the older strains, carries 2 copies of *sigH*, due to a second genomic duplication (DU2), and its expression is at least 2-fold higher [11]. SigH is an alternative extra-cytoplasmic sigma factor involved in the response to heat shock and oxidative stress, positively regulating the expression of other genes, including *dnaK *and possibly *groEL2 *[74]. GroEL2 (Rv0440, BCG0479; Hsp65) and DnaK (Rv0350, BCG0389; Hsp70) are chaperones involved in protein-folding, and have been associated with the induction of protection against TB infection in mice by immunization with experimental DNA vaccines [75,76]. Recently, these mycobacterial chaperones have been described as having vital moonlighting functions when present outside the cell: GroEL2 acts as a major adhesin, mediating binding of *Mtb *to monocytes and the soluble protein is capable of competing for this binding, reducing bacterial association to macrophages [77]. DnaK stimulates the secretion of chemokines required for granuloma formation [78] and its overexpression was found to favor the host over the pathogen during chronic *Mtb *infection [79]. All in all, subtle variations in the balance of expression and/or localization of these proteins may have profound impacts on the interaction between the bacteria (in this case, different BCG vaccine strains) and the host's immune system, impacting vaccine efficacy.

## Conclusions

The findings reported here provide new information about the proteomic characteristics of the BCG Moreau vaccine strain and contribute to shed more light on the differentiated immune response and the variable effectiveness of the different BCG vaccines. In Brazil, approximately 90,000 new cases of TB are reported annually by the health system [80]. The BCG Moreau vaccine has been used since 1925, and its production by Fundação Ataulpho de Paiva (FAP) currently represents 5% of the BCG vaccine production in the world [10]. According to recent data from the WHO, global BCG immunization increased since the 1980's and Brazil, with a population close to 200 million, shows over 99% coverage for BCG vaccination [81]. Despite the genetic differences accumulated in BCG strains, the originally described protective efficacy of BCG Moreau was not reduced, and the Brazilian strain is regarded as one of the most immunogenic among the vaccine preparations that are currently available [82,83]. The exploration of the secreted sub-proteome of *M. bovis *BCG Moreau provides valuable information regarding specific proteins, many of which have been implicated in protective immune responses, and helps defining candidates for future vaccination strategies.

## Methods

### Bacterial strains and growth conditions

*Mycobacterium bovis *BCG Pasteur 1173P2 was obtained from the Pasteur Institute (Paris, France) culture collection, and stocks were maintained at -80°C. *Mycobacterium bovis *BCG Moreau was provided by Fundação Ataulpho de Paiva (FAP). Both strains were cultured as surface pellicles, for 2 weeks at 37°C, in 100 ml of Sauton vaccine production medium, provided by FAP.

### Sample preparation

Culture filtrate proteins (CFPs) were obtained after separation of culture supernatants from the bacterial pellicles and subsequent centrifugation at 2,500 × g for 10 min at 4°C. The resulting supernatant was filtered through a 0.22 μm low protein binding membrane (Millipore Express; Millipore, Bedford, MA, USA) in order to remove any remaining bacteria. CFPs (on average 5.5 mg total protein) were precipitated with 17% (v/v) TCA and washed with cold acetone. Finally, proteins were dissolved in 1.5 ml of IEF buffer (8 M urea, 2% CHAPS, 4 mM tributylphosphine [TBP], 0.4% ampholytes pH 3-10) for 1 h at room temperature. Protein concentration was determined using the RC-DC Kit (Bio-Rad). Proteins were stored at -80°C until analysis.

### Two dimensional gel electrophoresis (2DE)

IPG strips and all 2DE reagents were purchased from Bio-Rad (Hercules, CA, USA). Isoelectric focusing was performed at 20°C on 17 cm IPG strips, using 500 μg of CFPs diluted in a final volume of 300 μl in rehydration buffer (8 M urea, 2% CHAPS, 4 mM TBP, 0.4% ampholytes pH 3-10). Samples were applied to IPG strips (pH intervals of 3-6, 4-7 and 5-8) by in-gel rehydration and incubated for 1 h at room temperature. Isoelectric focusing was performed on a Protean^® ^IEF cell (Bio-Rad) with maximum current of 50 μA/strip. Focusing parameters used for IPG strips in the pH range 4-7 and 5-8 were: active rehydration (50 V) for 11 h; step 1- linear gradient from 1 to 250 V over 20 min; step 2 - linear gradient from 250 to 10,000 V over 2 h; step 3- constant 10,000 V until 80,000 Vh was achieved. For IPG strips in the pH range 3-6, step 3 was constant 10,000 V until 60,000 Vh was achieved. After isoelectric focusing, proteins were reduced in 130 mM DTT and alkylated in 270 mM iodoacetamide, both in equilibration buffer (6 M urea, 2% SDS, 375 mM Tris-HCl pH 8.8, 20% glycerol). Second dimension separation was done in 17 cm, 12% or 15% SDS-PAGE gels, 1.0 mm thick, using a vertical system (Bio-Rad) in standard Laemli buffer [84] at 40 mA/gel, 10°C, until the tracking dye left the gel.

### Protein visualization and image analysis

Gels were stained with colloidal Coomassie Brilliant Blue G-250 essentially as described [85], and documented using a GS-800™ auto-calibrating imaging densitometer (Bio-Rad). Image analysis was performed using PDQuest™ software version 8.0.1 (Bio-Rad). Comparative 2DE data were derived from 4 separate protein preparations, each one obtained from independent cultures. The spots were quantified on the basis of their relative 'volume': the amount of a protein spot was expressed as the sum of the intensities of all the pixels that made up the spot. To compensate for subtle differences in sample loading, gel staining and de-staining, the volume of each spot was normalized in relation to the total density of valid spots present in the gel image. After automated detection and matching, manual editing was carried out. To determine the experimental pI and *M*_r _coordinates for each single protein spot, 2DE gels were calibrated using a selected set of five protein landmarks distributed throughout the gel.

### Protein digestion, peptide extraction and MS/MS analysis

In-gel digestion of 2DE separated protein spots was carried out essentially as described [86]. Briefly, protein spots were excised and the gel pieces washed 3 times with 50% (v/v) acetonitrile (ACN) in 25 mM ammonium bicarbonate for 15 min each, dehydrated in ACN, and dried in a vacuum centrifuge. Gel pieces were rehydrated in 15 μl of 50 mM ammonium bicarbonate containing 200 ng of sequencing grade modified trypsin (Promega). This step was performed for 40 minutes at 4°C and, after that, 20 μl of 50 mM ammonium bicarbonate were added to keep the gel pieces wet during tryptic digestion (37°C, 16 h). To extract peptides, 20 μl of 0.5% (v/v) trifluoroacetic acid (TFA) in 50% (v/v) ACN were added and samples were sonicated 3 times for 10 min each in a sonicator bath. The supernatant was recovered and concentrated under vacuum to a volume of approximately 10 μl. The resulting peptides were extracted, partially dried, and salts were removed using C18 ZipPlate (Millipore, Bedford, MA) following the manufacturer's instructions.

The tryptic peptides were analyzed in a 4700-Proteomics Analyzer MALDI-TOF/TOF (Applied Biosystems, Foster City, CA). All mass spectra were acquired on positive ion reflector mode with 2,000 shots per spot and externally mass calibrated with a peptide mixture. The 10 most intense ion peaks from the peptide mass fingerprinting (or MS run) were further submitted to fragmentation using PSD mode with CID gas off and 1 keV collision energy.

### Protein identification

Following MS acquisition, each spectrum was submitted to a peptide mass fingerprinting search, in the case of MS/MS spectra, using Mascot version 2.2 (Matrix Science - http://www.matrixscience.com/ ). For protein identification, the search was performed against the NCBI-nr non-redundant database (NCBI-nr200709, National Center for Biotechnology Information, http://www.ncbi.nlm.nih.gov/) without taxonomy restriction. When necessary, further searches were performed against the *Mycobacterium tuberculosis *database (http://genolist.pasteur.fr/tuberculist). Decoy search was performed automatically on the search form from Mascot using the same database described above with taxonomy restriction to the *Mycobacterium tuberculosis *complex to determine the false discovery rate (FDR). A FDR < 3.0% to peptide matches above homology or identity threshold was considered significant. For Mascot searches, the parameters used were trypsin as the enzyme of choice and one missed cleavage, ± 1 Da for the precursor mass, ± 0.5 Da for the fragment ion mass. Oxidation of methionines along with N-terminal acetylation of proteins, N-terminal formylation, deamidation and cyclization of glutamine (pyro-glutamate) were allowed as possible modifications whereas alkylation of cysteines (carbamidomethylcysteines) was set as constant modification. Identification was considered valid for Mascot protein scores greater than 30 and a significance threshold of p < 0.05. If a protein 'hit' was identified by only one peptide, the MS/MS data was to exhibit a clear spectrum with sequence tags that matched at least three consecutive y or b fragment ion series. Finally, a good correlation between the experimental and theoretical molecular mass and pI was also considered for positive identifications.

Putative signals for protein export were predicted using SignalP 3.0 (http://www.cbs.dtu.dk/services/SignalP/), LipoP 1.0 (http://www.cbs.dtu.dk/services/ LipoP/), TatP 1.0 (http://www.cbs.dtu.dk/services/TatP/) and SecretomeP 2.0 (http://www.cbs.dtu.dk/services/SecretomeP/). Potential transmembrane domais were predicted with TMHMM 2.0 (http://www.cbs.dtu.dk/services/TMHMM/).

Molecular weight (*M*_r_) and pI of secreted proteins was calculated with the Expasy compute pI/Mw tool (http://www.expasy.ch/tools/pi_tool.html).

### Statistical analysis

Spot intensity differences obtained from comparative 2DE gel images of *M. bovis *BCG strains Moreau and Pasteur were statistically analyzed by one-way ANOVA with Student's t-test to determine significant differences among group means. Statistical analysis was carried out using the data obtained from 4 different sets of independent biological samples. A p-value ≤0.05 was considered as statistically significant.

## Competing interests

The authors declare that they have no competing interests.

## Authors' contributions

MBP contributed in the experimental design, data acquisition and interpretation and was involved in writing the manuscript. DEK carried out the mass spectrometry analysis and protein identification. PCR and MPP contributed to data acquisition. LHFG carried out the PCR assays. RFS provided technical assistance. LRRCB contributed to data interpretation and manuscript revision. WMD took part in supervision, data interpretation and writing the manuscript. LML was responsible for the experimental design, supervision, data interpretation and writing the manuscript. All authors have read and approved the final manuscript.

## Supplementary Material

Additional file 1 **Figure S1 - PCR confirmation of the genetic identity of the BCG strains used**. Click here for file

Additional file 2 Table S1 - *M. bovis *BCG Moreau culture filtrate proteins identified by MS/MS Click here for file

Additional file 3 **Table S2 - Predicted localization of identified proteins**. Click here for file

Additional file 4 **Table S3 - *M***_**r **_**and pI of secreted proteins**. Click here for file

Additional file 5 **Figure S2 - Magnified 2DE gel regions showing protein spots differentially expressed between BCG strains Moreau and Pasteur**. Panels A - F represent the magnified gel regions indicated in Figure 4. Protein spot numbering is the same as in Figure 1. Click here for file

Additional file 6 **Figure S3 - Magnified 2DE gel regions showing protein spots expressed exclusively in BCG strains Moreau or Pasteur**. Panels A and B represent the magnified gel regions as indicated in Figure 4. Protein spot numbering is the same as in Figure 1. MPT64 (spots 69 and 158) and CFP21 (spot 96) are only found in BCG Moreau culture filtrate (panel A), while Rv3400 (BCG3470) was only found in BCG Pasteur (panel B). Click here for file
