# Supplementary material for: Proteomic profile of culture filtrate from the Brazilian vaccine strain Mycobacterium bovis BCG Moreau compared to M. bovis BCG Pasteur
Source: BMC Microbiol. 2011 Apr 20;11:80. doi: 10.1186/1471-2180-11-80 (PMC3094199; doi:10.1186/1471-2180-11-80)

Additional file 1, Figure S1: PCR confirmation of the genetic identity of BCG strains Pasteur (P) and Moreau (M), used in this study. PCR products were generated with primers flanking BCG Moreau specific deletions in genes *rv2930* (*fadD26;* PCR products of 1635 bp for BCG Pasteur and 695 bp for BCG Moreau) and *rv3887c* (products of 1667 bp for BCG Pasteur and 831 bp for BCG Moreau). Molecular size markers (Mw) indicated to the left, in bp.

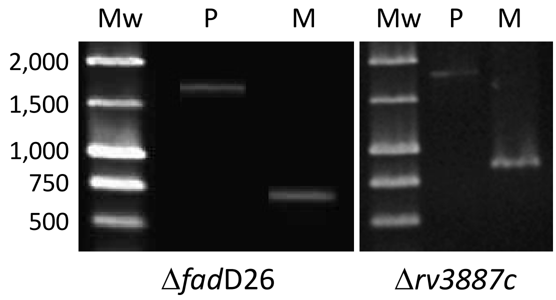

Supplement: Additional file 1 — Figure S1 - PCR confirmation of the genetic identity of the BCG strains used. [file 1471-2180-11-80-S1.PDF]
