# Supplementary material for: Proteomic profile of culture filtrate from the Brazilian vaccine strain Mycobacterium bovis BCG Moreau compared to M. bovis BCG Pasteur
Source: BMC Microbiol. 2011 Apr 20;11:80. doi: 10.1186/1471-2180-11-80 (PMC3094199; doi:10.1186/1471-2180-11-80)
Supplement: Additional file 2 — Table S1 - M. bovis BCG Moreau culture filtrate proteins identified by MS/MS [file 1471-2180-11-80-S2.PDF]

Additional file 2, Table S1. *M. bovis* BCG Moreau culture filtrate proteins identified by MS/MS

Spot no.	*M. tb.* H37Rv ortholog	BCG Pasteur ortholog	gene	Protein Identification	FC*	Protein score	Peptide Sequence	Delta[&] (Da)	Ion score	Cov. (%)	Theor. M_r (kDa)	Theor. pI	Exp. M_r (kDa)	Exp. pI
1	Rv1475c	BCG1537c	*acn*	Probable iron-regulated aconitate hydratase	7	355	AVIAESFER	-0.0400	45	7	102.45	4.72	84.43	5.28
							GDGATIEFDAVVR	-0.0506	64					
							AEPSIEIQYTPAR	-0.0558	59					
							TTIAPGSQVVNDYYDR	-0.0706	57					
							FVEFYGEGVAEVPLANR	-0.0823	138					
2	Rv0684	BCG0733	*fusA1*	Elongation factor G	2	79	LGETYDTVEIPADLAEQAEEYR	-0.1332	57	3	77.20	4.69	75.97	5.35
							IGADFYFSVR	-0.0779	22					
3	Rv1837c	BCG1872c	*glcB*	Probable malate synthase G	7	328	VVFINTGFLDR	-0.0500	72	8	80.40	4.83	74.58	5.40
							ISSQLLANWLR	-0.0470	55					
							ATIEQLLTIPLAK	-0.0535	50					
							NYTAPGGGQFTLPGR	-0.0555	72					
							VVADLTPQNQALLNAR	-0.0657	88					
4	Rv0350	BCG0389	*dnaK*	Probable chaperone protein; Hsp70	0	202	DAGQIAGLNVLR	0.0572	52	8	66.83	4.59	66.28	4.77
							LLGSFELTGIPPAPR	0.0778	48					
							SETFTTADDNQPSVQIQVYQGER	0.1262	106					
5	Rv0440	BCG0479	*groEL2*	60 kDa chaperonin 2	0	378	TIAYDEEAR	-0.0010	39	17	56.72	4.56	61.69	4.75
							GYISGYFVTDPER	0.0070	36					
							QIAFNSGLEPGVVAEK	0.0013	56					
							QEIENSDSDYDREK	0.0063	85					
							DETTIVEGAGDTDAIAGR	0.0050	41					
							KTDDVAGDGTTTATVLAQALVR	0.0134	129					
6	Rv0440	BCG0479	*groEL2*	60 kDa chaperonin 2	0	698	GYISGYFVTDPER	0.0149	54	20	56.72	4.56	59.30	4.70
							QIAFNSGLEPGVVAEK	0.0153	83					
							QEIENSDSDYDREK	0.0158	75					
							DETTIVEGAGDTDAIAGR	0.0158	96					
							KTDDVAGDGTTTATVLAQALVR	0.0278	209					
							TDDVAGDGTTTATVLAQALVR	0.0232	100					
							NLPAGHGLNAQTGVYEDLLAAGVADPVK	0.0403	92					
7	Rv2462c	BCG2482c	*tig*	Probable trigger factor protein	3	369	FNELLVEQGSSR	0.0465	84	25	50.63	4.17	57.80	4.40
							LIAGLDDAVVGLSADESR	0.0518	92					
							LAAGEHAGQEAQVTVTVR	0.0630	30					
							INVEVPFAELEPDFQR	0.0653	48					
							EYGQDLQFTAEVDIRPK[c]	-0.9212	54					
							VSAGEAEEAEPADEGAAR	0.0598	60					
8	Rv0020c	BCG0050c	*tb39.8*	Conserved hypothetical protein	10	355	LGHSEIIVR	-0.0646	69	20	56.00	4.64	52.28	4.50
							QDYGGGADYTR[e]	-0.0769	58					
							GGYPPETGGYPPQPGYPRPR	-0.1125	40					
							YTESPQVPGYAPQGGGYAEPAGR	-0.1274	101					
							HPGQGDYPEQIGYPDQGGYPEQR	-0.1159	85					

Additional file 2, Table S1. *(continued)*

Spot no.	*M. tb.* H37Rv ortholog	BCG Pasteur ortholog	gene	Protein Identification	FC*	Protein score	Peptide Sequence	Delta[&] (Da)	Ion score	Cov. (%)	Theor. M_r (kDa)	Theor. pI	Exp. M_r (kDa)	Exp. pI
9	Rv0020c	BCG0050c	*tb39.8*	Conserved hypothetical protein	10	227	GGYPPETGGYPPQPGYPRPR	-0.1125	19	16	56.00	4.64	52.28	4.54
							HPGQGDYPEQIGYPDQGGYPEQR	-0.1159	21					
							QDYGGGADYTR[e]	-0.0769	43					
							YTESPQVPGYAPQGGGYAEPAGR	-0.1274	103					
							LGHSEIIVR	-0.0646	47					
10	Rv0020c	BCG0050c	*tb39.8*	Conserved hypothetical protein	10	235	QDYGGGADYTR[e]	-0.0536	45	9	56.00	4.64	51.98	4.59
							YTESPQVPGYAPQGGGYAEPAGR	-0.0952	109					
							LGHSEIIVR	-0.0492	40					
11	Rv1860	BCG1896	*apa*	Alanine and proline rich secreted protein	3	92	TTGDPPFPGQPPPVANDTR	-0.0146	32	12	32.70	4.70	47.73	4.37
							INQETVSLDANGVSGSASYYEVK[c]	0.0248	65					
12	Rv1860	BCG1896	*apa*	Alanine and proline rich secreted protein	3	185	TTGDPPFPGQPPPVANDTR	-0.0797	77	17	32.70	4.70	48.30	4.20
							LGSDMGEFYMPYPGTR[a]	-0.0780	17					
							INQETVSLDANGVSGSASYYEVK[c]	-0.0638	96					
13	Rv1860	BCG1896	*apa*	Alanine and proline rich secreted protein	3	192	TTGDPPFPGQPPPVANDTR	-0.0782	84	17	32.70	4.70	42.91	4.23
							LGSDMGEFYMPYPGTR[a]	-0.0814	16					
							INQETVSLDANGVSGSASYYEVK[c]	-0.0826	96					
14	Rv1860	BCG1896	*apa*	Alanine and proline rich secreted protein	3	176	LGSDMGEFYMPYPGTR[a]	-0.0084	37	17	32.70	4.70	43.27	4.01
							TTGDPPFPGQPPPVANDTR	-0.0013	66					
							INQETVSLDANGVSGSASYYEVK[c]	0.0187	77					
15	Rv0577	BCG0622	*tb27.3*	Conserved hypothetical protein	10	125	AAAAGGQVIAEPADIPSVGR	-0.0441	125	7	27.34	4.20	27.76	4.41
16	Rv2007c	BCG2024c	*fdxA*	Probable ferredoxin	7	70	SCVQECPVDCIYEGAR[f]	-0.9593	45	14	12.06	3.96	24.60	3.80
							ACIEECPVDCIYEGAR[f]	0.0360	45					
17	Rv2220	BCG2237	*glnA1*	Glutamine synthetase	7	252	SVFDDGLAFDGSSIR	-0.1079	89	10	53.57	4.84	59.34	5.44
							GGYFPVAPNDQYVDLR	-0.1133	89					
							DGAPLMYDETGYAGLSDTAR[a]	-0.1337	74					
18	Rv3248c	BCG3277c	*saH*	S-adenosyl-L-homocysteine hydrolase	7	403	SIIVLSEGR	-0.0821	41	15	54.32	4.85	57.80	5.51
							IADLSLADFGR	-0.0892	58					
							GVTEETTTGVLR	-0.0935	51					
							NDEYDNEVYR	-0.1035	80					
							AGVVPPAEEDDPAEWK	-0.1280	61					
							EQAEYLGVDVEGPYKPDHYR	-0.1666	112					
19	Rv0234c	BCG0271c	*gabD1*	Probable succinate-semialdehyde dehydrogenase	7	208	FAAPALMAGNVGLLK[a]	-0.0521	34	18	54.30	5.22	56.93	5.40
							TFTAATDDEVDAAIAR	-0.0614	48					
							HASNVPQCALYLADVIAR[f]	-0.0585	34					
							YYAENAEALLADEPADAAK	-0.0710	64					
							FIVHADIYDDFVDKFVAR	-0.0914	36					
20	Rv0234c	BCG0271c	*gabD1*	Probable succinate-semialdehyde dehydrogenase	7	50	VGDPTDPDTDVGPLATEQGR	-0.1797	50	4	54.30	5.22	56.97	5.34
21	Rv0467	BCG0507	*icl*	Isocitrate lyase	7	145	DQPFITGER	-0.0453	63	13	47.09	4.79	52.69	5.34
							SAEQIQQEWDTNPR	-0.0369	31					
							IATTVDPNSSTTALTGSTEEGQFH	-0.0478	34					
							VLIPTQQHIR	-0.0368	28					

Additional file 2, Table S1. *(continued)*

Spot no.	*M. tb.* H37Rv ortholog	BCG Pasteur ortholog	gene	Protein Identification	FC*	Protein score	Peptide Sequence	Delta[&] (Da)	Ion score	Cov. (%)	Theor. M_r (kDa)	Theor. pI	Exp. M_r (kDa)	Exp. pI
22	Rv1098c	BCG1158c	*fum*	Fumarate hydratase	7	332	AVENFPISGR	-0.0078	46	15	50.14	5.18	53.45	5.78
							TAANSFEAQAAR	-0.0102	75					
							CIAGLTANVEHLR[f]	-0.0192	78					
							VVAVLVAQTGLSELR	-0.0079	54					
							LGELAIGGTAVGTGLNAPDDFGVR	0.0062	79					
23	Rv1098c	BCG1158c	*fum*	Fumarate hydratase	7	72	AVENFPISGR	0.0000	21	10	50.14	5.18	54.28	5.71
							LGELAIGGTAVGTGLNAPDDFGVR	-0.0538	26					
							VVAVLVAQTGLSELR	-1.0303	31					
24	Rv0462	BCG0502	*lpdC*	Alpha keto acid dehydrogenase complex	7	173	NYGVDVTIVEFLPR	-0.0405	79	11	49.24	5.70	54.44	5.98
							THYDVVVLGAGPGGYVAAIR	-0.0350	61					
							LVPGTSLSANVVTYEEQILSR	-0.0430	40					
26	Rv1093	BCG1153	*glyA1*	Probable serine hydroxymethiltransferase 1	7	211	VLDFAAFR	-0.0388	48	12	45.00	6.58	58.59	6.74
							VIIAGWSAYPR	-0.0536	59					
							LLGLDLANGGHLTHGMR[a.c]	-0.0753	23					
							YYGGCEHVDVVENLAR[f]	-0.0903	86					
27	Rv1133c	BCG1194c	*metE*	Probable homocysteine methyltransferase	7	267	ADQAEYLR	0.0020	33	11	81.58	4.92	76.30	5.71
							ATEGYWAGR	0.0117	46					
							AVDGAGAPIER	0.0039	29					
							AGEIDEAEYVR	0.0083	40					
							QPFTATITGSPR	0.0089	50					
							LGLDVLVHGEPER	0.0141	32					
							LHLPPLPTTTIGSYPQTSAIR	0.0350	46					
28	Rv0685	BCG0734	*tuf*	Probable iron-regulated elongation factor TU	2	230	AFDQIDNAPEER	-0.0777	85	15	43.59	5.12	49.94	5.82
							LLDQGQAGDNVGLLLR	-0.0821	50					
							ELLAAQEFDEDAPVVR	-0.0838	69					
							ADAVDDEELLELVEMEVR[a]	-0.0972	30					
							VLHDKFPDLNETK	-0.0891	39					
29	Rv0685	BCG0734	*tuf*	Probable iron-regulated elongation factor TU	2	160	HYAHVDAPGHADYIK	-0.0801	42	10	43.59	5.12	49.92	5.78
							VLHDKFPDLNETK	-0.0791	36					
							AFDQIDNAPEER	-0.0699	82					
30	Rv0685	BCG0734	*tuf*	Probable iron-regulated elongation factor TU	2	195	VLHDKFPDLNETK	-0.0891	39	10	43.59	5.12	49.32	5.73
							AFDQIDNAPEER	-0.0836	74					
							HYAHVDAPGHADYIK	-0.0955	82					
31	Rv0685	BCG0734	*tuf*	Probable iron-regulated elongation factor TU	2	111	AFDQIDNAPEER	-0.0706	68	7	43.59	5.12	49.68	5.65
							ELLAAQEFDEDAPVVR	-0.0935	47					
32	Rv1869c	BCG1905c	*rv1869c*	Probable reductase	7	163	EFLSFWLDGDSR	-0.0219	57	15	43.64	4.73	47.47	5.31
							SLSDFTIQTSDWYR	-0.0353	67					
33	Rv1323	BCG1385	*fadA4*	Probable acetyl-CoA acetyltransferase	1	132	ITLHAALQLAR	-0.0076	54	10	40.08	4.69	47.33	5.38
							ANTTAAALAGLKPAFR	-0.0035	40					
							DGVFADEVIPVNIPQR	-0.0013	37					

Additional file 2, Table S1. ***(continued)***

Spot no.	*M. tb.* H37Rv ortholog	BCG Pasteur ortholog	gene	Protein Identification	FC*	Protein score	Peptide Sequence	Delta[&] (Da)	Ion score	Cov. (%)	Theor. M_r (kDa)	Theor. pI	Exp. M_r (kDa)	Exp. pI
34	Rv1074c	BCG1132c	*fadA3*	Acetyl-CoA acetyltransferase	1	421	NPLFDGAQER	-0.0315	72	17	42.65	4.67	45.99	5.31
							SAAAAAGADEWHDPR	-0.0444	47					
							AGEGDAFISAGVETVSR	-0.0507	145					
							VVAVALGYDFLPGTTVNR	-0.0670	126					
							GNSDSWPDTKNPLFDGAQER	-0.0795	40					
35	Rv1436	BCG1497	*gap*	Probable glyceraldehyde-3-phosphate dehydrogenase (GAPDH)	7	100	VVSWYDNEWGYSNR	-0.0851	27	9	35.96	5.05	43.60	5.51
							LPCDVGLEGDDTIVVGR	-0.0659	47					
							VPIPTGSVTDLTVDLSTR	-0.0871	26					
36	Rv3045	BCG3069	*adhC*	Probable NADP-dependent alcohol dehydrogenase	7	201	HWNAGANTR[c]	-0.9676	32	15	37.07	4.93	41.62	5.67
							VGVGCFVDSCR[f]	0.0265	23					
							SYYATADPDTFR	0.0310	24					
							DGQPTQGGYSEAIVVDENYVLR[c]	-0.9100	125					
37	Rv1656	BCG1695	*argF*	Ornithine carbamoyltransferase	7	105	YVDAIVWR	-0.0512	53	8	33.06	5.06	42.76	5.70
							LLALADSDAIVLHCLPAHR[f]	-0.1139	55					
38	Rv1122	BCG1183	*gnd2*	Probable 6-phosphogluconate dehydrogenase	7	49	ESPDLAEFSGR	0.0021	22	8	36.36	5.10	41.18	5.76
							AEPIFATVAPGVAAAPR	-0.0059	27					
39	Rv0928	BCG0980	*pstS3*	Periplasmic phosphate-binding lipoprotein	3	390	SFQGGVGEGAR	-0.0562	71	23	37.95	5.85	39.50	5.50
							SDESGTTDNFQR	-0.0509	88					
							RPGSYPIVLATYEIVCSK[f]	-0.0767	40					
							IFNGSITQWNNPAIQALNR[c]	-0.0712	129					
							AFLQSTIGAGQSGLGDNGYIPIPDEFK[c]	-0.0897	62					
40	Rv0928	BCG0980	*pstS3*	Periplasmic phosphate-binding lipoprotein	3	247	IFNGSITQWNNPAIQALNR[c]	-0.0712	96	17	37.95	5.85	39.40	5.30
							SFQGGVGEGAR	-0.0562	46					
							RPGSYPIVLATYEIVCSK[f]	-0.0767	40					
							YLQAASNGAWGK[c]	-0.0660	65					
41	Rv1464	BCG1525	*csd*	Probable cysteine desulfurase	7	215	ASFAVYNTADEVDR	-0.0205	48	12	44.60	6.05	45.05	6.24
							ELVAAAIEGLSGIDGVR	-0.0201	75					
							NATEALNLVSYVLGDSR	-0.0181	58					
42	Rv2427c	BCG2444c	*proA*	Probable γ-glutamyl phosphate reductase	7	41	GLVDVVIPR	-0.0037	29	7	43.74	5.93	45.62	6.29
							TALVGLELPADAVQLLSAADR	0.0196	13					
43	Rv2145c	BCG2162c	*wag31*	Conserved hypothetical protein	3	377	TYLESQLEELGQR	-0.1048	58	20	28.28	4.52	34.27	5.26
							GSAAPVDSNADAGGFDQFNR	-0.1439	171					
							GYNEDEVDAFLDLVENELTR	-0.2014	154					
44	Rv2971	BCG2993	*rv2971*	Probable oxidoreductase	7	258	LATPDQGFTR	0.0083	27	24	30.34	4.51	31.83	4.77
							WNLQLGNAVVVR	-0.0043	27					
							YVDAWGGMIQSR	0.0057	60					
							LIDTAYAYGNEAAVGR	0.0104	111					
							ANAQHTVVTQSYCPLALGR[f]	0.0244	34					

Additional file 2, Table S1. ***(continued)***

Spot no.	*M. tb.* H37Rv ortholog	BCG Pasteur ortholog	gene	Protein Identification	FC*	Protein score	Peptide Sequence	Delta[&] (Da)	Ion score	Cov. (%)	Theor. M_r (kDa)	Theor. pI	Exp. M_r (kDa)	Exp. pI
45	Rv2971	BCG2993	*rv2971*	Probable oxidoreductase	7	334	LATPDQGFTR	0.0049	76	38	30.34	4.51	31.94	4.72
							WNLQLGNAVVVR	0.0052	93					
							YVDAWGGMIQSR	0.0022	46					
							VREDPLTYAGT	0.0097	30					
							LIDTAYAYGNEAAVGR	0.0138	118					
							ANAQHTVVTQSYCPLALGR[f]	0.0125	124					
							LLDNPTVTSIASEYVK	0.0006	104					
							AVSAALEIGCR	0.0068	49					
46	Rv2889c	BCG2910c	*tsf*	Probable elongation factor TSF	2	94	YLSRDDVPEDIVASER	-0.1227	44	12	28.78	5.01	33.46	5.46
							NALAETDGDFDKAVEALR	-0.1035	51					
47	Rv0815c	BCG0867c	*cysA2*	Probable thiosulfate sulfurtransferase	7	167	SSHTWFVLR	-0.0513	29	19	31.01	4.95	32.51	5.55
							DFVDAQQFSK	-0.0560	13					
							AFRDEVLAAINVK[c]	-1.0513	25					
							VVFVEVDEDTSAYDR	-0.0794	49					
							VVFVEVDEDTSAYDRDHIAGAIK	-0.1078	54					
48	Rv3389c	BCG3602	*rv3389c*	Double hotdog hydratase	7	129	VGTFNPAALLHGSQGIR	-0.0845	104	30	30.22	4.79	33.76	5.65
							ALVAELGGGVAANITSIAAR	-0.0882	151					
							FTKPVFPGETLSTVIWR	-0.0961	72					
							GCDPESGSLVAETLTTLVLR[f]	-0.0986	112					
49	Rv2334	BCG2356	*cysK1*	Probable cysteine synthase A	7	333	LEFFNPANSVK	-0.0584	63	23	32.75	4.93	34.30	5.70
							SIAEDITQLIGR	-0.0568	65					
							LIVVVLPDFGER	-0.0598	73					
							EEGLLVGISSGAATVAALQVAR	-0.1034	55					
50	Rv0363c	BCG0401c	*fba*	Probable fructose-biphosphate aldolase	7	171	YPVNVALHTDHCPK[f]	-0.1042	76	15	36.54	5.59	35.40	6.17
							LGLPADAKPFDFVFHGGSGSLK	-0.1439	74					
51	Rv1070c	BCG1128c	*echA8*	Probable enoyl-CoA hydratase	1	322	TYETILVER	-0.0098	62	27	27.27	4.67	25.98	5.46
							LGVLPGMGGSQR [a]	-0.0156	40					
							VVPADDLLTEAR	-0.0104	74					
							ATATTISQMSASAAR[a]	-0.0134	24					
							AFESSLSEGLLYER	-0.0115	100					
52	Rv3804c	BCG3866c	*fbpA*	Secreted antigen 85-A	1	118	ASDMWGPKEDPAWQR[a]	0.4464	31	15	35.69	6.51	30.60	5.80
							VWVYCGNGKPSDLGGNNLPAK[c,f]	0.5743	30					
							ALGATPNTGPAPQGA	0.3278	64					
53	Rv2277c	BCG2294c	*rv2277c*	Possible glycerolphosphodiesterase	7	45	QEGLQVAESR	-0.0152	47	3	31.85	4.92	30.37	5.71
54	Rv3804c	BCG3866c	*fbpA*	Secreted antigen 85-A	1	314	WETFLTSELPGWLQANR	-0.0815	58	25	35.69	6.51	30.90	5.60
							VQFQSGGANSPALYLLDGLR	-0.0841	141					
							ALGATPNTGPAPQGA	0.3278	64					
							ASDMWGPKEDPAWQR[a]	0.4464	31					
							VWVYCGNGKPSDLGGNNLPAK[c,f]	0.5743	30					
55	Rv3804c	BCG3866c	*fbpA*	Secreted antigen 85-A	1	180	VQFQSGGANSPALYLLDGLR	-0.0853	122	10	35.69	6.51	31.10	5.40
							WETFLTSELPGWLQANR	-0.0815	58					
56	Rv3846	BCG3909	*sodA*	Superoxide dismutase [FE]	0	42	AFWNVVNWADVQSR[c]	-0.9765	43	6	23.02	6.43	25.30	6.70

Additional file 2, Table S1. *(continued)*

Spot no.	*M. tb.* H37Rv ortholog	BCG Pasteur ortholog	gene	Protein Identification	FC*	Protein score	Peptide Sequence	Delta[&] (Da)	Ion score	Cov. (%)	Theor. M_r (kDa)	Theor. pI	Exp. M_r (kDa)	Exp. pI
57	Rv3804c	BCG3866c	*fbpA*	Secreted antigen 85-A	1	141	VQFQSGGANSPALYLLDGLR	-0.0841	141	6	35.69	6.51	31.03	5.24
58	Rv1886c	BCG1923c	*fbpB*	Secreted antigen 85-B	1	257	ASDMWGPSSDPAWQR[c]	-0.0160	47	20	34.55	5.72	29.57	5.27
							AADMWGPSSDPAWER[a]	-0.0160	63					
							WETLLTSELPQWLSANR	-0.0682	51					
							VQFQSGGNNSPAVYLLDGLR[c]	-1.0559	148					
59	Rv2716	BCG2729	*rv2716*	Conserved hypothetical protein	10	395	GSLIHTTWSPEGWVR	-0.0716	110	32	24.57	4.75	29.72	5.31
							MFAANLGVTEDEATGAAAIR	-0.0799	99					
							TEIPFAGHPTVGASWWLR	-0.1127	80					
							VFTDSDGNFGNPLGVINASK	-0.0841	106					
60	Rv0129c	BCG0163c	*fbpC*	Secreted antigen 85-C	1	116	FLEGLTLR	0.1049	44	7	36.77	6.27	26.37	4.72
							VQFQGGGPHAVYLLDGLR	0.2199	73					
61	Rv0129c	BCG0163c	*fbpC*	Secreted antigen 85-C	1	93	VQFQGGGPHAVYLLDGLR	-0.0191	58	7	36.77	6.27	26.30	4.83
							WETFLTR	-0.0406	37					
62	Rv3029c	BCG3052c	*fixA*	Probable electron transfer flavoprotein (beta-subunit)	7	112	QVPDTWSER[b.e]	0.0013	29	22	28.08	4.37	26.30	4.59
							VTDEGEGGNQIVQYLVAQK	0.9930	30					
							ETDEGVFTLEATLPAVISVNEK	1.0039	53					
63	Rv3724B	BCG3784	*cut5*	Probable cutinase	3	122	GTGEPPGIGSVGGLFVDALR	0.1046	46	18	23.48	6.08	25.65	5.22
							SLGVYAVNYPASNDFASSDFPK	0.0956	77					
64	Rv2110c	BCG2127c	*prcB*	Proteasome (beta subunit)	7	54	GIFPTAVIIDADGAVDVPESR	-0.0314	14	15	30.31	4.42	24.06	4.70
							VAVEALYDAADDDSATGGPDLVR	-0.0414	40					
65	Rv0148	BCG0184	*rv0148*	Probable short-chain type dehydrogenase/reductase	7	290	TALDEFGAVHGVVSNAGILR	-0.0213	108	37	29.78	5.12	29.10	5.72
							EYALTLAGEGASVVVNDLGGAR	-0.0199	44					
							VVVATSTSGLFGNFGQTNYGAAK	-0.0070	35					
							VALFGNDGANFDKPPSVQDVAAR	-0.0090	71					
66	Rv2773c	BCG2790c	*dapB*	Dihydrodipicolinate reductase	7	212	TSFVPGVLLAVR	-0.0587	45	25	25.73	5.75	29.74	6.14
							GADVDGIPVHAVR	-0.0561	45					
							FFDSAEVIELHHPHK	-0.0673	61					
							LAGLVAHQEVLFGTEGEILTIR	-0.0974	62					
67	Rv3803c	BCG3865c	*fbpD*	Secreted MPT51 antigen	1	84	MFYNQYR[a]	-0.0713	29	13	31.08	6.63	25.65	5.22
							QWDTFLSAELPDWLAANR[c]	-1.0863	61					
68	Rv2534c	BCG2556c	*efp*	Probable elongation factor P	2	64	SSAGTKPATLQTGAQINVPLFINTGDK[c]	-0.0873	23	29	20.41	5.65	26.10	6.10
							DGSDFVFMDSQDYEQHPLPEALVGDAAR[a]	0.9203	41					
69	Rv1980c[1]	deleted	*mpt64*	Immunogenic protein MPT64	3	62	AFDWDQAYR	-0.0254	24	12	24.82	4.60	21.00	4.50
							EAPYELNITSATYQSAIPPR	-0.1203	42					
70	Rv3036c	BCG3060c	*tb22.2*	Probable conserved secreted protein	3	75	DGFVNVAQGSPLR	0.0316	49	12	24.40	5.06	25.76	4.72
							FFQDLGGAHPSTWYK	0.0296	26					
71	Rv2301	BCG2317	*cut2*	Probable cutinase	3	42	AAAACPDVQVVFAR[h]	-0.9606	42	5	23.92	5.10	24.40	5.04
72	Rv3036c	BCG3060c	*tb22.2*	Probable conserved secreted protein	3	133	DGFVNVAQGSPLR	0.0571	65	22	24.40	5.06	25.76	5.28
							FFQDLGGAHPSTWYK	0.0810	39					
73	Rv1732c	BCG1771c	*rv1732c*	Conserved hypothetical protein	10	37	GQLDDSRPGNGRPVTAADVR[c]	-0.1139	40	10	19.39	4.87	25.98	5.38

Additional file 2, Table S1. ***(continued)***

Spot no.	*M. tb.* H37Rv ortholog	BCG Pasteur ortholog	gene	Protein Identification	FC*	Protein score	Peptide Sequence	Delta[&] (Da)	Ion score	Cov. (%)	Theor. M_r (kDa)	Theor. pI	Exp. M_r (kDa)	Exp. pI
74	Rv0733	BCG0783	*adk*	Probable adenylate kinase	3	118	GRADDTDDVILNR	-0.0339	60	22	20.12	4.76	25.90	5.44
							TVDAVGTMDEVFAR[a]	-0.0342	41					
							NIEEGTKLGVEAKR	-0.0348	22					
75	Rv0787	BCG0839	*rv0787*	Hypothetical protein	10	280	WPAMAGAAVAVTR[b]	-0.1084	44	20	34.16	5.56	26.31	5.81
							FGFEQVCATPAFPHDSR[f]	-0.1375	84					
							ACQLGAPLQSPSVTDDEPTR[c,f]	-1.1371	157					
76	Rv0632c	BCG0679c	*echA3*	Probable enoyl-CoA hydratase	1	116	SAYQQATGLAK	-0.1018	23	16	24.39	5.62	26.24	5.92
							DDVGALVITGNGR[c]	-0.1075	66					
							EFAGLNQHAHAATK	-0.1385	34					
77	Rv2882c	BCG2903c	*frr*	Ribosome recycling factor	2	140	TTHQYVTQIDELVK	-0.0971	45	17	20.84	5.77	26.20	6.31
							HKEGELLEV	-0.0043	47					
							VAVPQLTEER	0.0027	50					
79	Rv2429	BCG2448	*ahpD*	Alkyl hydroperoxide reductase subunit D	0	154	NPQVLADIGAEATDHLSAAAR[c]	-0.9640	90	23	18.78	7.06	23.64	7.04
							SSVLDQEQLWGTLLASAAATR	0.0062	68					
80	Rv1827	BCG1862	*cfp17*	Conserved hypothetical protein	10	275	DQPYQMDATSEQHSSGQPPQATR[a]	0.0868	29	45	17.25	4.06	19.33	3.89
							FLLDQAITSAGR	-0.0001	74					
							HPDSDIFLDDVTVSR	0.0010	96					
							LENNEFNVVDVGSLNGTYVNR[c]	-0.9969	88					
							TDMNPDIEKDQTSDEVTVETTSVFR[c,g]	0.2133	17					
81	Rv1827	BCG1862	*cfp17*	Conserved hypothetical protein	10	63	LENNEFNVVDVGSLNGTYVNR[c]	-0.8853	26	21	17.25	4.06	19.10	3.93
							HPDSDIFLDDVTVSR	0.0710	37					
82	Rv1827	BCG1862	*cfp17*	Conserved hypothetical protein	10	35	HPDSDIFLDDVTVSR	-0.0226	35	9	17.25	4.06	19.17	3.99
83	Rv0054	BCG0085	*ssb*	Probable single-strand binding protein	2	132	TVIEVEVDEIGPSLR	-0.0386	54	32	17.35	4.84	21.50	5.56
							FTPSGAAVANFTVASTPR	-0.0554	49					
84	Rv2140c	BCG2157c	*tb18.6*	Conserved hypothetical protein	10	219	YYVAVHAVK	-0.0478	37	23	18.63	5.52	19.48	5.33
							WSGFPSETR	-0.0051	38					
							AVIFGTYEQR	0.0001	61					
							YVGAAPPPGHGVHR	-0.0062	86					
85	Rv0054	BCG0085	*ssb*	Probable single-strand binding protein	2	290	AGDTTITIVGNLTADPELR	0.0992	90	36	17.35	4.84	21.30	5.37
							FTPSGAAVANFTVASTPR	0.0912	75					
							DGEALFLR	0.0290	32					
							TVIEVEVDEIGPSLR	0.0896	93					
86	Rv2140c	BCG2157c	*tb18.6*	Conserved hypothetical protein	10	253	WSGFPSETR	-0.0334	45	32	18.63	5.52	19.90	5.56
							ELPGGALTLVNDAGMR	-0.0279	35					
							YVGAAPPPGHGVHR	-0.0279	78					
							YYVAVHAVK	-0.0380	31					
							AVIFGTYEQR	-0.0283	64					
87	Rv0398c	BCG0435c	*rv0398c*	Possible secreted protein	3	48	VLGCQQNTAGSGAGFGAR[c]	1.0600	50	8	21.65	6.45	21.19	5.80
88	Rv2140c	BCG2157c	*tb18.6*	Conserved hypothetical protein	10	159	YVGAAPPPGHGVHR	0.0509	68	18	18.63	5.52	19.80	5.81
							YYVAVHAVK	0.0221	36					
							AVIFGTYEQR	0.0371	55					

Additional file 2, Table S1. ***(continued)***

Spot no.	*M. tb.* H37Rv ortholog	BCG Pasteur ortholog	gene	Protein Identification	FC*	Protein score	Peptide Sequence	Delta[&] (Da)	Ion score	Cov. (%)	Theor. M_r (kDa)	Theor. pI	Exp. M_r (kDa)	Exp. pI
89	Rv0009	BCG0009	*ppiA*	Probable iron-regulated peptidyl-prolyl cis-trans isomerase A	2	299	IALFGNHAPK	-0.0527	42	42	19.24	6.23	22.17	6.11
							TVANFVGLAQGTK[c]	-0.0667	31					
							HTIFGEVIDAESQR	-0.0712	72					
							VIQGFMIQGGDPTGTGR[a]	-0.0800	49					
							DYSTQNASGGPSGPFYDGAVFHR	-0.0985	114					
90	Rv0009	BCG0009	*ppiA*	Probable iron-regulated peptidyl-prolyl cis-trans isomerase A	2	323	IALFGNHAPK	0.0044	32	53	19.24	6.23	21.43	6.39
							TVANFVGLAQGTK[c]	-0.9807	36					
							DYSTQNASGGPSGPFYDGAVFHR	0.0296	126					
							VIQGFMIQGGDPTGTGR[a]	0.0115	57					
							HTIFGEVIDAESQR	0.0112	58					
							TATDGNDRPTDPVVIESITIS	0.0242	23					
91	Rv0009	BCG0009	*ppiA*	Probable iron-regulated peptidyl-prolyl cis-trans isomerase A	2	88	DYSTQNASGGPSGPFYDGAVFHR	-0.0763	41	20	19.24	6.23	22.10	6.73
							HTIFGEVIDAESQR	-0.0613	51					
92	Rv0009	BCG0009	*ppiA*	Probable iron-regulated peptidyl-prolyl cis-trans isomerase A	2	40	IALFGNHAPK	0.0050	16	12	19.24	6.23	22.20	6.90
							TVANFVGLAQGTK	0.0037	28					
93	Rv2875	BCG2897	*mpt70*	Major secreted immunogenic protein	3	143	LPASTIDELK	-0.0362	30	14	19.07	4.54	22.50	4.00
							QTLQGASVTVTGQGNSLK	-0.0577	113					
94	Rv2875	BCG2897	*mpt70*	Major secreted immunogenic protein	3	258	TNSSLLTSILTYHVVAGQTSPANVVGTR	-0.0747	145	23	19.07	4.54	22.50	4.00
							QTLQGASVTVTGQGNSLK	-0.0577	113					
94	Rv2873	BCG2895	*mpt83*	Cell surface lipoprotein MPT83	3	153	LLSSILTYHVIAGQASPSR	-0.0388	17	17	22.07	4.73	21.80	4.20
							IDGTHQTLQGADLTVIGAR	0.0313	140					
95	Rv2875	BCG2897	*mpt70*	Major secreted immunogenic protein	3	356	LPASTIDELK	-0.0261	61	28	19.07	4.54	23.05	4.40
							QTLQGASVTVTGQGNSLK	-0.0341	154					
							TNSSLLTSILTYHVVAGQTSPANVVGTR	-0.0747	145					
96	Rv1984c[1]	deleted	*cfp21*	Probable cutinase precursor	3	32	SIGVYAVNYPASDDYR	0.1565	32	7	21.76	5.52	16.60	4.55
97	Rv2244	BCG2261	*acpM*	Meromycolate extension acyl carrier protein	1	35	IPDEDLAGLR	0.0300	35	8	12.52	3.74	16.50	3.77
98	Rv3418c	BCG3488c	*groES*	10 kDa chaperonin	0	31	YNGEEYLILSAR	-0.0161	31	12	10.80	4.34	12.20	3.91
99	Rv3418c	BCG3488c	*groES*	10 kDa chaperonin	0	51	YNGEEYLILSAR	-0.0099	51	12	10.80	4.34	15.41	4.30
100	Rv3418c	BCG3488c	*groES*	10 kDa chaperonin	0	227	EKPQEGTVVAVGPGR	-0.0421	87	31	10.80	4.34	15.40	4.31
							RIPLDVAEGDTVIYSK	-0.0460	32					
							IPLDVAEGDTVIYSK	-0.0440	108					
101	Rv3418c	BCG3488c	*groES*	10 kDa chaperonin	0	620	YNGEEYLILSAR	-0.0352	95	73	10.80	4.34	12.32	4.43
							EKPQEGTVVAVGPGR	0.0600	100					
							IPLDVAEGDTVIYSK	-0.0265	124					
							RIPLDVAEGDTVIYSK	-0.0656	93					
							YGGTEIKYNGEEYLILSAR	-0.0640	53					
							ILVQANEAETTTASGLVIPDTAK	-0.0872	153					
102	Rv3418c	BCG3488c	*groES*	10 kDa chaperonin	0	293	EKPQEGTVVAVGPGR	-0.0395	112	31	10.80	4.34	12.01	4.63
							RIPLDVAEGDTVIYSK	-0.0474	63					
							IPLDVAEGDTVIYSK	-0.0437	118					

Additional file 2, Table S1. ***(continued)***

Spot no.	*M. tb.* H37Rv ortholog	BCG Pasteur ortholog	gene	Protein Identification	FC*	Protein score	Peptide Sequence	Delta[&] (Da)	Ion score	Cov. (%)	Theor. M_r (kDa)	Theor. pI	Exp. M_r (kDa)	Exp. pI
103	Rv2031c	BCG2050c	*hspX*	Heat shock protein (α−crystallin homolog)	0	82	SEFAYGSFVR	-0.0263	46	17	16.23	4.75	13.54	4.51
							AELPGVDPDKDVDIMVR	-0.0375	36					
107	Rv3418c	BCG3488c	*groES*	10 kDa chaperonin	0	465	YNGEEYLILSAR	-0.9608	88	73	10.80	4.34	8.33	4.58
							EKPQEGTVVAVGPGR	0.0161	108					
							IPLDVAEGDTVIYSK	-0.0046	22					
							RIPLDVAEGDTVIYSK	0.0075	40					
							YGGTEIKYNGEEYLILSAR	0.0021	130					
							ILVQANEAETTTASGLVIPDTAK	0.0180	86					
108	Rv0652	BCG0701	*rplL*	Probable 50S ribosomal protein L7/L12	2	33	DLVDGAPKPLLEK	0.0300	33	10	13.44	4.30	16.56	4.51
109	Rv1926c	BCG1965c	*mpt63*	Immunogenic protein MPT63	3	216	GSVTPAVSQFNAR	-0.0328	99	23	16.51	4.67	15.31	4.65
							VLWQAAGPDTISGATIPQGEQSTGK	-0.0688	117					
110	Rv0164	BCG0200	*tb18.5*	Conserved hypothetical protein	10	56	YPEWNEGVK	-0.0001	58	5	17.74	4.62	15.31	4.65
111	Rv1926c	BCG1965c	*mpt63*	Immunogenic protein MPT63	3	303	GSVTPAVSQFNAR	-0.0195	83	51	16.51	4.67	16.50	4.47
							LGSELTMTDTVGQVVLGWK	-0.0386	50					
							SSTAVIPGYPVAGQVWEATATVNAIR	-0.0478	29					
							VLWQAAGPDTISGATIPQGEQSTGK	-0.0312	141					
112	Rv1926c	BCG1965c	*mpt63*	Immunogenic protein MPT63	3	84	VLWQAAGPDTISGATIPQGEQSTGK	-0.9964	84	15	16.51	4.67	15.61	4.47
113	Rv2878c	BCG2900c	*mpt53*	Soluble secreted antigen MPT53 precursor	3	288	ADVGAMQSFVSK[a]	-0.0318	57	20	18.37	5.20	14.40	4.52
							YNVPWQPAFVFYR[b]	-0.0338	53					
							YNLNFTNLNDADGVIWAR	-0.0345	79					
							ADGTSTFVNNPTAAMSQDELSGR[a]	-0.0208	100					
114	Rv2031c	BCG2050c	*hspX*	Heat shock protein (alpha-crystallin homolog)	0	181	SEFAYGSFVR	-0.0313	64	32	16.23	4.75	13.93	4.59
							AELPGVDPDKDVDIMVR	-0.0557	60					
							TVSLPVGADEDDIKATYDK	-0.0543	57					
115	Rv2031c	BCG2050c	*hspX*	Heat shock protein (alpha-crystallin homolog)	0	172	SEFAYGSFVR	-0.0476	43	32	16.23	4.75	13.40	4.83
							AELPGVDPDKDVDIMVR	-0.0789	63					
							TVSLPVGADEDDIKATYDK	-0.0886	66					
116	Rv2031c	BCG2050c	*hspX*	Heat shock protein (alpha-crystallin homolog)	0	55	SEFAYGSFVR	-0.0421	28	17	16.23	4.75	13.62	4.99
							AELPGVDPDKDVDIMVR	-0.0850	27					
117	Rv2031c	BCG2050c	*hspX*	Heat shock protein (alpha-crystallin homolog)	0	158	SEFAYGSFVR	-0.0030	59	32	16.23	4.75	13.04	4.58
							AELPGVDPDKDVDIMVR	-0.0190	35					
							TVSLPVGADEDDIKATYDK	-0.0143	64					
118	Rv1038c	BCG1096c	*esxJ*	ESAT-6 like protein 2	3	274	NIVNMLHGVR[a,j]	-0.0338	50	46	10.99	5.02	13.62	4.70
							FEVHAQTVEDEAR[j]	0.0000	110					
							FEVHAQTVEDEARR[j]	-0.0605	105					
							FMTDPHAMR[a,j]	-0.0042	19					
119	Rv1793	BCG1825	*esxN*	Putative ESAT-6 like protein 5	3	186	DVLAAGDFWGGAGSVACQEFITQLGR[f,k]	0.0640	105	43	9.94	4.56	11.97	4.73
							NFQVIYEQANAHGQK[l]	0.0945	73					
120	Rv3208A	BCG3235c	*tb9.4*	Conserved hypothetical protein	10	119	IAYVEIGVADAR	0.0621	49	35	9.43	4.77	10.98	5.10
							ELVFSSAQTPSEVEELVSNALR	0.0909	72					

Additional file 2, Table S1. *(continued)*

Spot no.	*M. tb.* H37Rv ortholog	BCG Pasteur ortholog	gene	Protein Identification	FC*	Protein score	Peptide Sequence	Delta[&] (Da)	Ion score	Cov. (%)	Theor. M_r (kDa)	Theor. pI	Exp. M_r (kDa)	Exp. pI
121	Rv3648c	BCG3706c	*cspA*	Probable cold shock protein A	0	109	VEFEIGHSPK	0.0400	61	39	7.37	4.96	9.37	5.48
							TLEENQKVEFEIGHSPK[c]	1.0400	48					
122	Rv3628	BCG3686	*ppa*	Inorganic pyrophosphatase	7	107	HFFVHYK	-0.0229	43	20	18.33	4.52	11.97	5.68
							MQFDVTIEIPK[a]	-0.0300	33					
							AADWVDRAEAEAEVQR	-0.0248	31					
123	Rv2376c	BCG2390c	*cfp2*	Low molecular weight antigen CFP2	3	115	GSLVEGGIGGTEAR	-0.0009	123	41	16.62	6.04	13.14	6.73
124	Rv3699	BCG3758	*rv3699*	Conserved hypothetical protein	10	234	TDEVMDWDSAYR[db]	-0.0258	25	32	25.00	4.44	24.06	4.65
							GLTTASFVQADITEFAAYPAGSAGR[c]	-0.9664	97					
							FSTVIDSTLFHSLPVDSR	0.0008	97					
							GAFPAELEVKPNEVDEDELR	-0.0186	135					
125	Rv1037c	BCG1095c	*esxI*	Putative ESAT-6 like protein 1	3	199	TINYQFGDVDAHGAMIR[d, l]	0.0400	108	2	9.82	4.48	10.77	4.55
							NFQVIYEQANAHGQK[l]	0.0300	111					
126	Rv1793	BCG1825	*esxN*	Putative ESAT-6 like protein 5	3	214	AQAASLEAEHQAIVR[l]	-0.0289	120	50	9.94	4.56	10.98	4.43
							NFQVIYEQANAHGQK[l]	-0.0244	74					
							TINYQFGDVDAHGAMIR[a,d,l]	-0.0342	22					
127	Rv1758	BCG1798	*cut1*	Probable cutinase	3	58	GTGEPPGVGGIGEDFIDALR	0.0100	58	13	22.00	4.03	21.00	3.90
128	Rv2626c	BCG2653c	*rv2626c*	Conserved hypothetical protein	10	39	GLAAGLDPNTATAGELAR	0.0300	41	12	15.52	4.77	14.38	5.33
129	Rv3369	BCG3441	*rv3369*	Conserved hypothetical protein	10	68	FGLTEAIAAYSTR	0.0200	68	9	15.72	5.69	14.67	5.37
130	Rv1038c	BCG1096c	*esxJ*	ESAT-6 like protein 2	3	57	NIVNMLHGVR[a,j]	-0.0043	35	24	10.99	5.02	14.67	5.37
							FEVHAQTVEDEARR[j]	-0.9988	22					
131	Rv2445c	BCG2465c	*ndkA*	Probable nucleoside diphosphate kinase	7	146	TLVLIKPDGIER	0.0200	55	22	14.51	5.18	14.64	5.61
							QLAGGTDPVQAAAPGTIR	-0.0200	147					
132	Rv3110	BCG3135	*moaB1*	Probable pterin-4-alpha-carbinolamine dehydratase	7	190	AEEVNHHPDIDIR	-0.0749	100	31	14.56	5.99	13.40	5.98
							VAERAEEVNHHPDIDIR	-0.0918	74				13.40	5.76
133	Rv1636	BCG1674	*tb15.3*	Iron-regulated conserved hypothetical protein	10	176	VTGTAPIYEILHDAK	-0.0117	83	20	15.31	5.62	16.46	6.00
							LIIASAYLPQHEDAR	-0.0405	93					
134	Rv3678c	BCG3736c	*rv3678c*	Conserved hypothetical protein	10	97	LGADVNPEEGKTLAR	0.3025	48	21	15.25	4.83	18.53	5.69
							TGNLVYTAGQLPLEAGK	0.3412	52					
137	Rv1911c	BCG1950c	*lppC*	Probable lipoprotein	3	40	QGYFGPCPPAGTGTHHYR[f]	-0.0395	40	8	19.78	5.76	23.05	4.83
140	Rv0884c	BCG0936c	*serC*	Possible phosphoserine aminotransferase	7	273	SLHLTYGEFSAK	-0.0220	62	13	40.23	4.53	47.47	4.72
							ADQLTPHLEIPTAIKPR	-0.0284	98					
							SQVVGTIDFVDDVDAGTVAK	-0.0278	113					
141	Rv0884c	BCG0936c	*serC*	Possible phosphoserine aminotransferase	7	225	SLHLTYGEFSAK	0.0330	62	13	40.23	4.53	47.47	4.77
							ADQLTPHLEIPTAIKPR	0.0071	70					
							SQVVGTIDFVDDVDAGTVAK	0.0000	93					
146	Rv3914	BCG3972	*trxC*	Thioredoxin (MPT46)	7	60	LDVDTNPETAR	-0.0263	62	9	12.54	4.82	14.08	4.76
148	Rv3592	BCG3657	*tb11.2*	Conserved hypothetical protein	10	94	INAIEVPAGAGPELEK	0.0421	38	16	11.18	6.35	11.97	6.00
							INAIEVPAGAGPELEKR	0.0525	56					
150	Rv0287	BCG0327	*esxG*	ESAT-6 like protein(TB9.8)	3	138	SLLDAHIPQLVASQSAFAAK[m]	0.1001	109	52	9.78	6.51	11.97	7.04
							HTIGQAEQAAMSAQAFHQGESSAAFQAAHAR[b,m]	0.1710	31					
151	Rv1636	BCG1674	*tb15.3*	Iron-regulated conserved hypothetical protein	10	132	VTGTAPIYEILHDAK	-0.0438	55	20	15.31	5.62	14.42	6.34
							LIIASAYLPQHEDAR	-0.0392	77					

Additional file 2, Table S1. ***(continued)***

Spot no.	*M. tb.* H37Rv ortholog	BCG Pasteur ortholog	gene	Protein Identification	FC*	Protein score	Peptide Sequence	Delta[&] (Da)	Ion score	Cov. (%)	Theor. M_r (kDa)	Theor. pI	Exp. M_r (kDa)	Exp. pI
152	Rv3803c	BCG3865c	*fbpD*	Secreted MPT51 antigen	1	50	APYENLMVPSPSMGR[a]	0.0925	36	6	31.08	6.63	27.00	6.00
							QWDTFLSAELPDWLAANR	-0.0874	50					
153	Rv1299	BCG1359	*prfA*	Probable peptide chain release factor	2	124	LAPIVATHR	-0.0233	13	13	39.04	4.85	47.33	5.51
							TYNFPENR	-0.0181	18					
							VGELDAQLTDMLAPR[a]	-0.0198	14					
							SGEGGEESALFAADLAR	-0.0129	82					
154	Rv1093	BCG1153	*glyA1*	Probable serine hydroxymethiltransferase 1	7	154	VLDFAAFR	0.0030	34	10	45.00	6.58	52.69	6.34
							VIIAGWSAYPR	0.0033	23					
							LLGLDLANGGHLTHGMR[a.c]	0.0036	40					
							YYGGCEHVDVVENLAR[f]	0.0016	57					
155	Rv1093	BCG1153	*glyA1*	Probable serine hydroxymethiltransferase 1	7	162	VLDFAAFR	0.0055	40	10	45.00	6.58	52.69	6.39
							VIIAGWSAYPR	0.0007	19					
							LLGLDLANGGHLTHGMR[a.c]	0.0005	56					
							YYGGCEHVDVVENLAR[f]	0.0148	46					
156	Rv1093	BCG1153	*glyA1*	Probable serine hydroxymethiltransferase 1	7	30	VLDFAAFR	0.0213	30	1	45.00	6.58	52.69	6.90
158	Rv1980c[1]	deleted	*mpt64*	Immunogenic protein MPT64	3	230	AFDWDQAYR	-0.0648	56	20	24.82	4.60	21.33	4.32
							SLENYIAQTR	-0.0642	42					
							DKFLSAATSSTPR	-0.0717	61					
							VYQNAGGTHPTTTYK	-0.0683	71					
159	Rv0125	BCG0159	*pepA*	Probable secreted serine protease	7	164	TQDVAVLQLR	0.0045	71	20	34.93	4.91	27.88	4.71
							SGGGSPTVHIGPTAFLGLGVVDNNGNGAR[c]	0.0207	65					
							GAGGLPSAAIGGGVAVGEPVVAMGNSGGQGGTPR[a]	0.0041	29					
160	Rv1926c	BCG1965c	*mpt63*	Immunogenic protein MPT63	3	261	TADGINYR	0.0127	31	28	16.51	4.67	16.61	4.65
							GSVTPAVSQFNAR	-0.0532	97					
							VLWQAAGPDTISGATIPQGEQSTGK	-0.0964	134					
161	Rv2220	BCG2237	*glnA1*	Glutamine synthetase	7	251	SVFDDGLAFDGSSIR	-0.1061	93	10	53.57	4.84	59.27	5.42
							GGYFPVAPNDQYVDLR	-0.1142	62					
							DGAPLMYDETGYAGLSDTAR	-0.1296	96					
162	Rv0063	BCG0094	*rv0063*	Possible oxidoreductase	7	291	YFGPNLSR	-0.0051	42	16	49.30	8.31	39.40	6.90
							VLQPDDGPQFATAK	0.0244	26					
							QLPGDINYDATTGR	0.0048	69					
							AYSVGGYVNYLEVNQPPAR	0.0137	93					
							QVFNTNYNGYTPAVIVTPTSQLDVQK[c]	0.0348	61					
163	Rv0216	BCG0253	*rv0216*	Conserved hypothetical protein	10	92	LVLDFYR	0.0012	25	8	35.79	7.00	35.40	6.90
							VGGPYFDDLSK	0.0119	22					
							FPAVGDTLYTR	0.0190	45					
164	Rv2376c	BCG2390c	*cfp2*	Low molecular weight antigen CFP2	3	262	GSLVEGGIGGTEAR	-0.0009	123	41	16.62	6.04	13.40	5.38
							LSSPVTQNVTFVNQGGWMLSR[a]	-0.0128	32					
							AAEHGDLPLSFSVTNIQPAAAGSATADVSVSGPK	-0.0170	109					

Additional file 2, Table S1. *(continued)*

Spot no.	*M. tb.* H37Rv ortholog	BCG Pasteur ortholog	gene	Protein Identification	FC*	Protein score	Peptide Sequence	Delta[&] (Da)	Ion score	Cov. (%)	Theor. M_r (kDa)	Theor. pI	Exp. M_r (kDa)	Exp. pI
165	Rv2971	BCG2993	*rv2971*	Probable oxidoreductase	7	56	WNLQLGNAVVVR	-0.0472	32	9	30.34	4.51	31.94	4.69
							LIDTAYAYGNEAAVGR	0.0120	47					
							LATPDQGFTR	-0.0274	17					
166	Rv0125	BCG0159	*pepA*	Probable secreted serine protease	7	43	TQDVAVLQLR	-0.0069	43	3	34.93	4.91	33.46	3.98
167	Rv0125	BCG0159	*pepA*	Probable secreted serine protease	7	56	TQDVAVLQLR	-0.0223	56	3	34.93	4.91	27.86	4.59
168	Rv0078A	BCG0110c	*rv0078A*	Hypothetical protein	10	66	ALVDAASPQDIQDAR	-0.0058	68	7	21.61	4.95	27.00	6.70
169	Rv1098c	BCG1158c	*fum*	Fumarate hydratase	7	75	LGELAIGGTAVGTGLNAPDDFGVR	-0.0524	44	8	50.14	5.18	54.31	5.62
							VVAVLVAQTGLSELR	-1.0303	31					
170	Rv1323	BCG1385	*fadA4*	Probable acetyl-CoA acetyltransferase	1	262	ITLHAALQLAR	-0.0341	113	10	40.08	4.69	47.33	5.34
							ANTTAAALAGLKPAFR	-0.0530	81					
							DGVFADEVIPVNIPQR	-0.0613	76					
171	Rv1323	BCG1385	*fadA4*	Probable acetyl-CoA acetyltransferase	1	424	APHLLMNSR[a]	-0.0043	19	19	40.08	4.69	47.33	5.40
							ITLHAALQLAR	-0.0004	79					
							ANTTAAALAGLKPAFR	-0.0014	58					
							DGVFADEVIPVNIPQR	0.0051	126					
							GSGVGVAALCGAGGQGDALILR[f]	0.0040	145					
172	Rv1626	BCG1664	*rv1626*	Probable two-component system transcriptional regulator	9	106	IAPIVVLTAFSQR	-0.0640	54	13	22.67	4.74	30.15	5.36
							EITALEGEVATLSER	-0.0748	53					
BCG[2]	Rv3400[2]	BCG3470	*rv3400*	Probable hydrolase	7	239	DDGAQVFDGSR	-0.0354	37	31	28.23	5.71	29.57	5.73
							AGNFAVVVGINR	-0.0243	63					
							LLRDDGAQVFDGSR	-0.0331	29					
							EEHIAGKPAPDSFLR	-0.0287	49					
							FVPFDPAADYHTYVDGK	-0.0170	38					
							AIEIPDGSPDDPGAAETVYGLGNR	-0.0235	24					

[1]Proteins identified only in *M. bovis* BCG Moreau.
[2]Protein identified only in *M. bovis* BCG Pasteur.
*Biological functions were obtained from Tuberculist (http://genolist.pasteur.fr/TubercuList/).
[&]Difference (error) between the experimental and calculated relative peptide molecular masses.
[a]Methionine modified by oxidation.
[b]Tryptophan modified by oxidation
[c]Peptide with asparagine or glutamine by deamidation.
[d]Peptide with acetylation on N-terminal threonine .
[e]Peptide with N-terminal modification of glutamine to pyroglutamic acid.
[f]Cysteine modified by carbamidomethyl.
[g]Peptide with N-terminal formylation.
[h]Peptide sequence shows 100% identity and positivity to the cutinase precursor, Cut4 of *Mycobacterium ulcerans* Agy99 (gi|118616630) and 78% identity and 85% positivity to the cutinase precursor, Cut2 (Rv2301) of *M. tuberculosis* (http://genolist.pasteur.fr/TubercuList/).
[i]Peptide sequence identified shows 100% identity and positivity to the putative ESAT-6 like protein (Rv1793).
[j]Peptide sequences show 100% identity and positivity to all these proteins: ESAT-6 like proteins EsxJ (Rv1038c), EsxK (Rv1197), EsxM (Rv1792), EsxP (Rv2347c) and EsxW (Rv3620c)
[k]Peptide sequence shows 100% identity and positivity to the putative ESAT-6 like protein EsxN (Rv1793) and putative ESAT-6 like protein EsxO (Rv2346c).
[l]Peptide sequences matched all these proteins: putative ESAT-6 like protein EsxI (Rv1037c), EsxL (Rv1198), EsxN (Rv1793), EsxO(Rv2346c) and EsxV (Rv3619c) .
[m]Peptide sequence shows 100% identity and positivity to the ESAT-6 like protein EsxG (TB9.8, Rv0287).
