# Supplementary material for: Proteomic profile of culture filtrate from the Brazilian vaccine strain Mycobacterium bovis BCG Moreau compared to M. bovis BCG Pasteur
Source: BMC Microbiol. 2011 Apr 20;11:80. doi: 10.1186/1471-2180-11-80 (PMC3094199; doi:10.1186/1471-2180-11-80)
Supplement: Additional file 3 — Table S2 - Predicted localization of identified proteins. [file 1471-2180-11-80-S3.PDF]

Additional file 3, Table S2: Predicted localization of identified proteins. Signal P (sec-dependent secretion), LipoP (lipoprotein signal peptide), TatP (twin-arginine translocation) and SecretomeP (non-classical secretion) tools were used for prediction of protein localization; number of potential transmembrane domais predicted with TMHMM. Proteins listed as secreted via the type VII secretion system [49] are indicated. Our data was compared to two previous studies on the culture filtrate proteome of *M. tuberculosis* H37Rv: the Max Planck 2DE database (http://web.mpiib-berlin.mpg.de/) and the work by de Souza *et al.,* 2008 [34].

M.tb. ortholog	BCG Pasteur ortholog	gene	Protein Identification	Signal P	LipoP	TatP	Secretome P	TMHMM	Type VII	MP-2DE	de Souza 2008
Rv0009	BCG0009	*ppiA*	Probable peptidyl-prolyl cis-trans isomerase A	–	–	–	yes	0	–	yes	yes
Rv0020c	BCG0050c	*tb39.8*	Conserved hypothetical protein	–	–	–	yes	0	–	yes	yes
Rv0054	BCG0085	*ssb*	Probable single-strand binding protein	–	–	–	yes	0	–	yes	yes
Rv0063	BCG0094	*rv0063*	Possible oxidoreductase	31-32	–	34-35	–	0	–	–	yes
Rv0078A	BCG0110c	*rv0078A*	Hypothetical protein	–	–	–	–	0	–	–	yes
Rv0125	BCG0159	*pepA*	Probable secreted serine protease	32-33	–	32-33	yes	1	–	yes	yes
Rv0129c	BCG0163c	*fbpC*	Secreted antigen 85-C	46-47	–	46-47	yes	1	–	yes	yes
Rv0148	BCG0184	*rv0148*	Probable short-chain dehydrogenase/reductase	–	–	–	–	0	–	–	yes
Rv0164	BCG0200	*tb18.5*	Conserved hypothetical protein	–	–	–	–	0	–	yes	yes
Rv0216	BCG0253	*rv0216*	Conserved hypothetical protein	–	–	–	–	0	–	–	yes
Rv0234c	BCG0271c	*gabD1*	Probable succinate-semialdehyde dehydrogenase	–	–	–	–	0	–	–	yes
Rv0287	BCG0327	*esxG*	ESAT-6 like protein(TB9.8)	–	–	–	–	0	yes	yes	yes
Rv0350	BCG0389	*dnaK*	Probable chaperone protein; Hsp70	–	–	–	–	0	–	yes	yes
Rv0363c	BCG0401c	*fba*	Probable fructose-biphosphate aldolase	–	–	–	–	0	–	yes	yes
Rv0398c	BCG0435c	*rv0398c*	Possible secreted protein	28-29	–	–	yes	1	–	–	yes
Rv0440	BCG0479	*groEL2*	60 kDa chaperonin 2	–	–	–	–	0	–	yes	yes
Rv0462	BCG0502	*lpdC*	Alpha keto acid dehydrogenase complex	–	–	–	–	0	–	yes	yes
Rv0467	BCG0507	*icl*	Isocitrate lyase	–	–	–	–	0	–	yes	yes
Rv0577	BCG0622	*tb27.3*	Conserved hypothetical protein	–	–	–	–	0	–	–	yes
Rv0632c	BCG0679c	*echA3*	Probable enoyl-CoA hydratase	–	–	–	–	0	–	–	yes
Rv0652	BCG0701	*rplL*	Probable 50S ribosomal protein L7/L12	–	–	–	–	0	–	–	–
Rv0684	BCG0733	*fusA1*	Elongation factor G	–	–	–	–	0	–	–	yes
Rv0685	BCG0734	*tuf*	Probable iron-regulated elongation factor TU	–	–	–	–	0	–	yes	–
Rv0733	BCG0783	*adk*	Probable adenylate kinase	–	–	–	–	0	–	yes	yes
Rv0787	BCG0839	*rv0787*	Hypothetical protein	–	–	–	–	0	–	–	yes
Rv0815c	BCG0867c	*cysA2*	Probable thiosulfate sulfurtransferase	–	–	–	yes	0	–	yes	–
Rv0884c	BCG0936c	*serC*	Possible phosphoserine aminotransferase	–	–	–	–	0	–	–	yes
Rv0928	BCG0980	*pstS3*	Periplasmic phosphate-binding lipoprotein	41-42	22-23	–	yes	0	–	–	yes
Rv1037c	BCG1095c	*esxI*	Putative ESAT-6 like protein 1	–	–	–	–	0	yes	–	yes
Rv1038c	BCG1096c	*esxJ*	ESAT-6 like protein 2	–	–	–	–	0	yes	–	yes
Rv1070c	BCG1128c	*echA8*	Probable enoyl-CoA hydratase	–	–	–	–	0	–	–	yes
Rv1074c	BCG1132c	*fadA3*	Acetyl-CoA acetyltransferase	–	–	–	–	0	–	yes	yes
Rv1093	BCG1153	*glyA1*	Probable serine hydroxymethiltransferase 1	–	–	–	–	0	–	yes	yes
Rv1098c	BCG1158c	*fum*	Fumarate hydratase	–	–	–	–	0	–	yes	yes

Additional file 3, Table S2: (continued)

M.tb. ortholog	BCG Pasteur ortholog	gene	Protein Identification	Signal P	LipoP	TatP	Secretome P	TMHMM	Type VII	MP-2DE	de Souza 2008
Rv1122	BCG1183	*gnd2*	Probable 6-phosphogluconate dehydrogenase	–	–	–	–	0	–	–	yes
Rv1133c	BCG1194c	*metE*	Probable homocysteine methyltransferase	–	–	–	–	0	–	–	yes
Rv1299	BCG1359	*prfA*	Probable peptide chain release factor 1	–	–	–	–	0	–	–	–
Rv1323	BCG1385	*fadA4*	Probable acetyl-CoA acetyltransferase	–	–	–	–	0	–	yes	yes
Rv1436	BCG1497	*gap*	Probable glyceraldehyde-3-P dehydrogenase	–	–	–	–	0	–	yes	yes
Rv1464	BCG1525	*csd*	Probable cysteine desulfurase	–	–	–	–	0	–	–	yes
Rv1475c	BCG1537c	*acn*	Probable iron-regulated aconitate hydratase	–	–	–	–	0	–	–	yes
Rv1626	BCG1664	*rv1626*	Probable 2-component transcriptional regulator	–	–	–	–	0	–	–	–
Rv1636	BCG1674	*tb15.3*	Iron-regulated conserved hypothetical protein	–	–	–	–	0	–	yes	–
Rv1656	BCG1695	*argF*	Ornithine carbamoyltransferase	–	–	–	–	0	–	–	yes
Rv1732c	BCG1771c	*rv1732c*	Conserved hypothetical protein	25-26	–	–	–	0	–	yes	–
Rv1758	BCG1798	*cut1*	Probable cutinase Culp5	–	–	–	–	0	–	–	–
Rv1793	BCG1825	*esxN*	Putative ESAT-6 like protein 5	–	–	–	–	0	yes	–	–
Rv1827	BCG1862	*cfp17*	Conserved hypothetical protein	–	–	–	yes	0	–	yes	yes
Rv1837c	BCG1872c	*glcB*	Probable malate synthase G	–	–	–	–	0	–	–	yes
Rv1860	BCG1896	*apa*	Alanine and proline rich secreted protein	39-40	–	–	yes	1	–	yes	yes
Rv1869c	BCG1905c	*rv1869c*	Probable reductase	–	–	–	–	0	–	–	yes
Rv1886c	BCG1923c	*fbpB*	Secreted antigen 85-B	40-41	–	40-41	yes	1	–	yes	yes
Rv1911c	BCG1950c	*lppC*	Probable lipoprotein	31-32	23-24	–	yes	0	–	–	yes
Rv1926c	BCG1965c	*mpt63*	Immunogenic protein MPT63	29-30	–	–	yes	1	–	yes	yes
Rv1980c	deleted	*mpt64*	Immunogenic protein MPT64	23-24	–	–	yes	1	–	yes	yes
Rv1984c	deleted	*cfp21*	Probable cutinase precursor	32-33	–	–	–	0	–	–	yes
Rv2007c	BCG2024c	*fdxA*	Probable ferredoxin	–	–	–	–	0	–	–	–
Rv2031c	BCG2050c	*hspX*	Heat shock protein (alpha-crystallin homolog)	–	–	–	–	0	–	yes	yes
Rv2110c	BCG2127c	*prcB*	Proteasome (beta subunit)	–	–	–	–	0	–	yes	yes
Rv2140c	BCG2157c	*tb18.6*	Conserved hypothetical protein	–	–	–	yes	0	–	yes	yes
Rv2145c	BCG2162c	*wag31*	Conserved hypothetical protein	–	–	–	–	0	–	yes	–
Rv2220	BCG2237	*glnA1*	Glutamine synthetase	–	–	–	yes	0	–	yes	yes
Rv2244	BCG2261	*acpM*	Meromycolate extension acyl carrier protein	–	–	–	–	0	–	–	yes
Rv2277c	BCG2294c	*rv2277c*	Possible glycerolphosphodiesterase	–	–	–	–	0	–	–	–
Rv2301	BCG2317	*cut2*	Probable cutinase	32-33	–	–	yes	1	–	yes	yes
Rv2334	BCG2356	*cysK1*	Probable cysteine synthase A	–	–	–	–	0	–	yes	yes
Rv2376c	BCG2390c	*cfp2*	Low molecular weight antigen CFP2	29-30	–	–	yes	1	–	–	yes
Rv2427c	BCG2444c	*proA*	Probable gamma-glutamyl phosphate reductase	–	–	–	–	0	–	–	yes
Rv2429	BCG2448	*ahpD*	Alkyl hydroperoxide reductase subunit D	–	–	–	–	0	–	–	yes
Rv2445c	BCG2465c	*ndkA*	Probable nucleoside diphosphate kinase	–	–	–	–	0	–	yes	yes
Rv2462c	BCG2482c	*tig*	Probable trigger factor protein	–	–	–	–	0	–	yes	–
Rv2534c	BCG2556c	*efp*	Probable elongation factor P	–	–	–	yes	0	–	yes	–

Additional file 3, Table S2: (continued)

M.tb. ortholog	BCG Pasteur ortholog	gene	Protein Identification	Signal P	LipoP	TatP	Secretome P	TMHMM	Type VII	MP-2DE	de Souza 2008
Rv2626c	BCG2653c	*rv2626c*	Conserved hypothetical protein	–	–	–	–	0	–	yes	–
Rv2716	BCG2729	*rv2716*	Conserved hypothetical protein	–	–	–	–	0	–	yes	yes
Rv2773c	BCG2790c	*dapB*	Dihydrodipicolinate reductase	–	–	–	–	0	–	yes	–
Rv2873	BCG2895	*mpt83*	Cell surface lipoprotein MPT83	53-54	24-25	–	yes	0	–	–	yes
Rv2875	BCG2897	*mpt70*	Major secreted immunogenic protein MPT70	30-31	–	–	yes	1	–	–	yes
Rv2878c	BCG2900c	*mpt53*	Soluble secreted antigen MPT53 precursor	37-38	–	–	yes	1	–	–	yes
Rv2882c	BCG2903c	*frr*	Ribosome recycling factor	–	–	–	–	0	–	yes	yes
Rv2889c	BCG2910c	*tsf*	Probable elongation factor TSF	–	–	–	–	0	–	yes	yes
Rv2971	BCG2993	*rv2971*	Probable oxidoreductase	–	–	–	–	0	–	–	yes
Rv3029c	BCG3052c	*fixA*	Probable electron transfer flavoprotein	–	–	–	–	0	–	yes	–
Rv3036c	BCG3060c	*tb22.2*	Probable conserved secreted protein	36-37	–	–	yes	0	–	yes	–
Rv3045	BCG3069	*adhC*	Probable NADP-dep.alcohol dehydrogenase	–	–	–	–	0	–	–	yes
Rv3110	BCG3135	*moaB1*	Probable pterin-4-alpha-carbinolamine dehydratase	–	–	–	–	0	–	–	–
Rv3208A	BCG3235c	*tb9.4*	Conserved hypothetical protein	–	–	–	–	0	–	yes	yes
Rv3248c	BCG3277c	*sahH*	S-adenosyl-L-homocysteine hydrolase	–	–	–	–	0	–	–	yes
Rv3369	BCG3441	*rv3369*	Conserved hypothetical protein	–	–	–	–	0	–	–	yes
Rv3389c	BCG3602	*rv3389c*	Double hotdog hydratase	–	–	–	–	0	–	yes	yes
Rv3400	BCG3470	*rv3400*	Probable hydrolase	–	–	–	–	0	–	yes	–
Rv3418c	BCG3488c	*groES*	10 kDa chaperonin	–	–	–	yes	0	–	yes	yes
Rv3592	BCG3657	*tb11.2*	Conserved hypothetical protein	–	–	–	–	0	–	yes	–
Rv3628	BCG3686	*ppa*	Inorganic pyrophosphatase	–	–	–	–	0	–	yes	–
Rv3648c	BCG3706c	*cspA*	Probable cold shock protein A	–	–	–	–	0	–	yes	–
Rv3678c	BCG3736c	*rv3678c*	Conserved hypothetical protein	–	–	–	–	0	–	yes	yes
Rv3699	BCG3758	*rv3699*	Conserved hypothetical protein	–	–	–	–	0	–	yes	–
Rv3724	BCG3784	*cut5*	Probable cutinase	35-36	–	–	–	0	–	–	yes
Rv3803c	BCG3865c	*fbpD*	Secreted MPT51 antigen	33-34	–	33-34	yes	1	–	yes	yes
Rv3804c	BCG3866c	*fbpA*	Secreted antigen 85-A	43-44	–	41-42	yes	1	–	yes	yes
Rv3846	BCG3909	*sodA*	Superoxide dismutase [FE]	–	–	–	–	0	–	yes	yes
Rv3914	BCG3972	*trxC*	Thioredoxin (MPT46)	–	–	–	–	0	–	yes	yes

Predictions using Signal P, LipoP, TatP, Secretome P and TMHMM, all at http://www.cbs.dtu.dk/services/. Numbers indicate residues flanking cleavage site prediction.
