# Supplementary material for: Proteomic profile of culture filtrate from the Brazilian vaccine strain Mycobacterium bovis BCG Moreau compared to M. bovis BCG Pasteur
Source: BMC Microbiol. 2011 Apr 20;11:80. doi: 10.1186/1471-2180-11-80 (PMC3094199; doi:10.1186/1471-2180-11-80)
Supplement: Additional file 4 — Table S3 - Mr and pI of secreted proteins. [file 1471-2180-11-80-S4.PDF]

Additional file 4, Table S3: M_r and pI of secreted proteins.

Mtb ortholog	BCG Pasteur ortholog	gene	Signal pep cleavage (Signal P)	Theor. M_r (kDa)*	Theor. pI*	Spot number	Exp. M_r (kDa)	Exp. pI
Rv0063	BCG0094	*rv0063*	31-32	49.30<br>(46.21)	8.32<br>(6.81)	162	39.40	6.90
Rv0125	BCG0159	*pepA*	32-33	34.93<br>(31.50)	5.04<br>(4.53)	159<br>166<br>167	27.88<br>33.46<br>27.86	4.71<br>3.98<br>4.59
Rv0129c	BCG0163c	*fbpC*	46-47	36.77<br>(32.02)	5.92<br>(4.99)	60<br>61	26.37<br>26.30	4.72<br>4.83
Rv0398c	BCG0435c	*rv0398c*	28-29	21.65<br>(19.16)	6.22<br>(5.36)	87	21.19	5.80
Rv0928	BCG0980	*pstS3*	41-42	37.95<br>(34.16)	5.75<br>(5.75)	39<br>40	39.50<br>39.40	5.50<br>5.30
Rv1732c	BCG1771c	*rv1732c*	25-26	19.40<br>(16.94)	5.02<br>(5.21)	73	25.98	5.38
Rv1860	BCG1896	*apa*	39-40	32.70<br>(28.74)	4.93<br>(4.52)	11<br>12<br>13<br>14	47.73<br>48.30<br>42.91<br>43.27	4.37<br>4.20<br>4.23<br>4.01
Rv1886c	BCG1923c	*fbpB*	40-41	34.54<br>(30.62)	5.62<br>(4.87)	58	29.57	5.27
Rv1911c	BCG1950c	*lppC*	31-32	19.80<br>(16.97)	5.76<br>(5.59)	137	23.05	4.83
Rv1926c	BCG1965c	*mpt63*	29-30	16.51<br>(13.65)	4.92<br>(4.50)	109<br>111<br>112<br>160	15.31<br>16.50<br>15.61<br>16.61	4.65<br>4.47<br>4.47<br>4.65
Rv1980c	deleted	*mpt64*	23-24	24.82<br>(22.43)	4.84<br>(4.60)	69<br>158	21.00<br>21.33	4.50<br>4.32
Rv1984c	deleted	*cfp21*	32-33	21.78<br>(18.66)	5.52<br>(4.69)	96	16.60	4.55
Rv2277c	BCG2294c	*rv2277c*	20-21	31.85<br>(30.06)	5.07<br>(5.18)	53	30.37	5.71
Rv2301	BCG2317	*cut2*	32-33	23.93<br>(20.62)	5.17<br>(4.96)	71	24.40	5.04
Rv2376c	BCG2390c	*cfp2*	29-30	16.63<br>(14.00)	6.04<br>(5.10)	123<br>164	13.14<br>13.40	6.73<br>5.38
Rv2873	BCG2895	*mpt83*	53-54	22.07<br>(17.02)	4.86<br>(4.48)	94	21.80	4.20
Rv2875	BCG2897	*mpt70*	30-31	19.07<br>(16.31)	4.75<br>(4.31)	93<br>94<br>95	22.50<br>21.80<br>23.05	4.00<br>4.20<br>4.40
Rv2878c	BCG2900c	*mpt53*	37-38	18.38<br>(14.62)	5.19<br>(4.57)	113	14.40	4.52
Rv3036c	BCG3060c	*tb22.2*	36-37	24.41<br>(20.94)	5.11<br>(4.91)	70<br>72	25.76<br>25.76	4.72<br>5.28
Rv3724	BCG3784	*cut5*	35-36	23.48<br>(19.91)	5.70<br>(4.89)	63	25.65	5.22
Rv3803c	BCG3865c	*fbpD*	33-34	31.09<br>(27.80)	6.13<br>(5.51)	67<br>152	25.65<br>27.00	5.22<br>6.00
Rv3804c	BCG3866c	*fbpA*	43-44	35.69<br>(31.65)	6.08<br>(5.32)	52<br>54<br>55<br>57	30.60<br>30.90<br>31.10<br>31.03	5.80<br>5.60<br>5.40<br>5.24

*Molecular weight and pI for full protein and, in parenthesis, for mature protein after removal of signal peptide region.
