# Supplementary figures and images for: Proteomic profile of culture filtrate from the Brazilian vaccine strain Mycobacterium bovis BCG Moreau compared to M. bovis BCG Pasteur
Source: BMC Microbiol. 2011 Apr 20;11:80. doi: 10.1186/1471-2180-11-80 (PMC3094199; doi:10.1186/1471-2180-11-80)

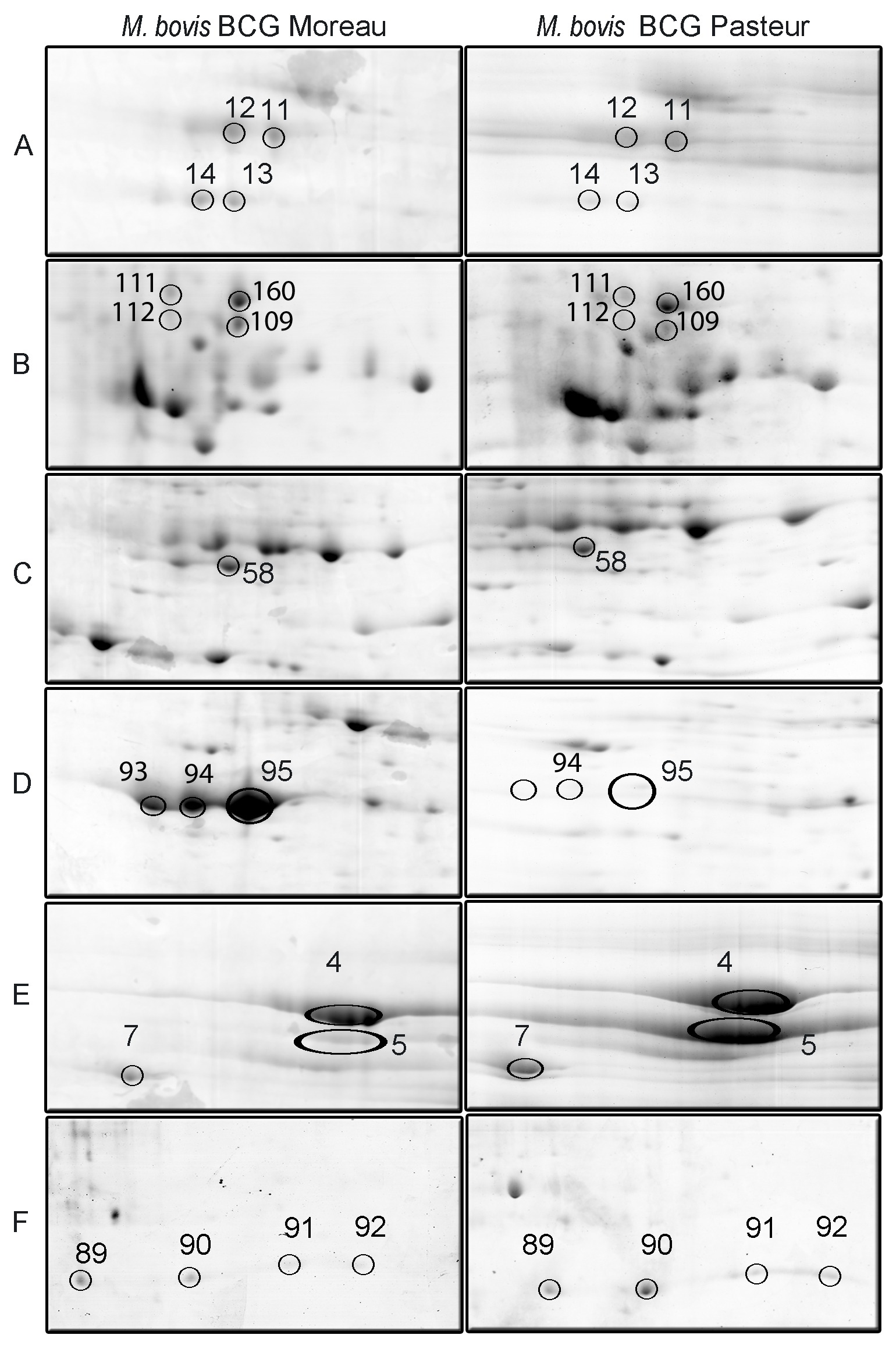

Supplement: Additional file 5 — Figure S2 - Magnified 2DE gel regions showing protein spots differentially expressed between BCG strains Moreau and Pasteur. Panels A - F represent the magnified gel regions indicated in Figure 4. Protein spot numbering is the same as in Figure 1. [file 1471-2180-11-80-S5.JPEG]

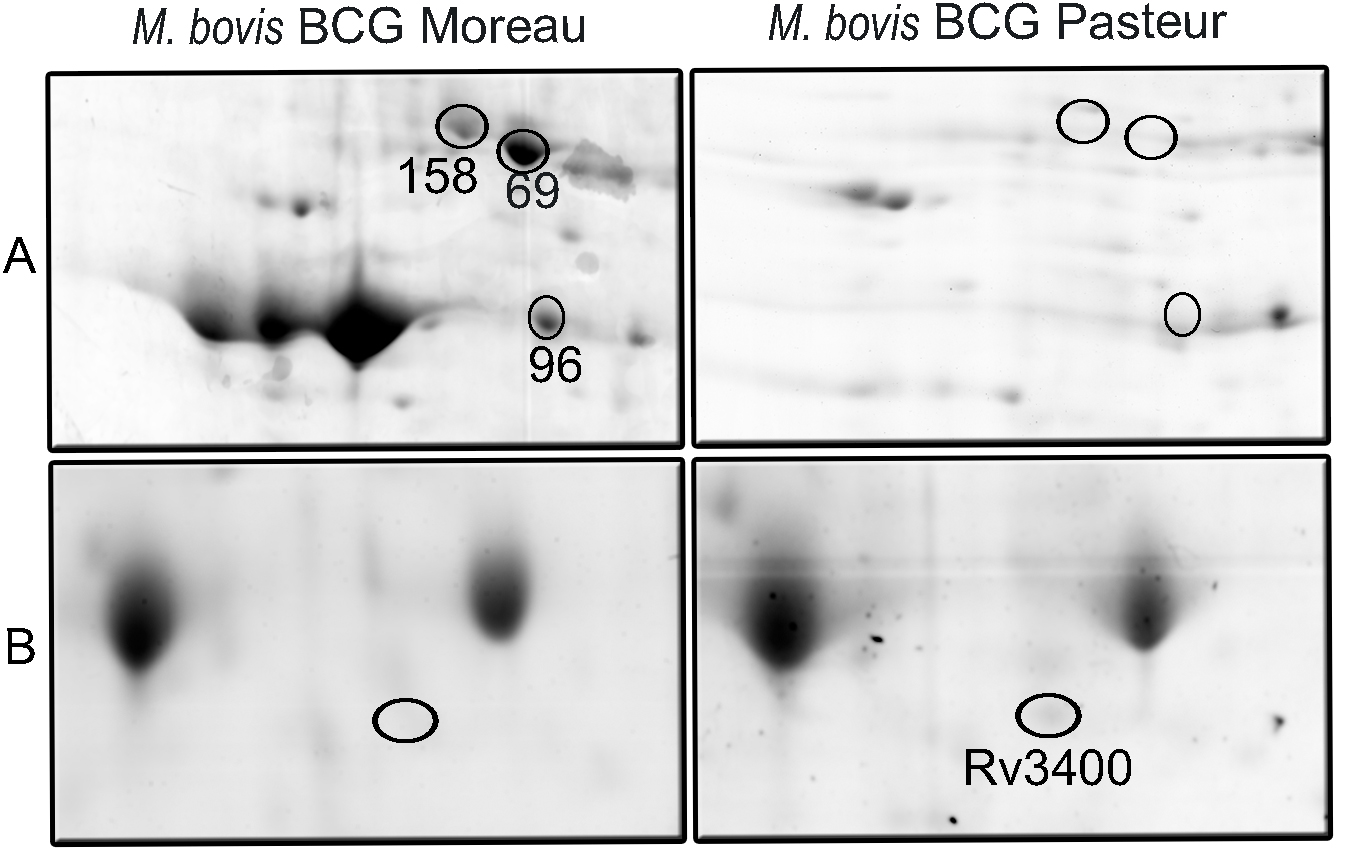

Supplement: Additional file 6 — Figure S3 - Magnified 2DE gel regions showing protein spots expressed exclusively in BCG strains Moreau or Pasteur. Panels A and B represent the magnified gel regions as indicated in Figure 4. Protein spot numbering is the same as in Figure 1. MPT64 (spots 69 and 158) and CFP21 (spot 96) are only found in BCG Moreau culture filtrate (panel A), while Rv3400 (BCG3470) was only found in BCG Pasteur (panel B). [file 1471-2180-11-80-S6.JPEG]
